# Comparison of Shifts of Potential Distributions in *Gleditsia* (Fabaceae) Between Eastern Asia and Eastern North America

**DOI:** 10.1002/ece3.72591

**Published:** 2025-11-29

**Authors:** Zhao‐Yu Yan, Hai‐Yang Wu, Bin Tian, Jun‐Wei Ye

**Affiliations:** ^1^ Yunnan Key Laboratory of Plateau Wetland Conservation, Restoration and Ecological Services Southwest Forestry University Kunming China; ^2^ Shangri‐La Potatso National Park Bita Lake Plateau Wetland Ecosystem Observation and Research Station of Yunnan Province Kunming Yunnan China; ^3^ College of Agronomy and Life Sciences Zhaotong University Zhaotong China; ^4^ Key Laboratory of Forest Resources Conservation and Utilization in the Southwest Mountains of China, Ministry of Education/Key Laboratory for Conserving Wildlife With Small Populations in Yunnan/College of Forestry Southwest Forestry University Kunming China

**Keywords:** background test, diversity anomaly, environment heterogeneity, last glacial maximum, MaxEnt, niche identity test, soil

## Abstract

The mechanism underlying the uneven distribution of biodiversity has attracted considerable interest. In Eastern Asia (EAS) and Eastern North America (ENA), a more heterogeneous environment in the EAS is a key factor explaining the uneven distributed biodiversity. To test the hypothesis, shifts of potential distributions of *Gleditsia* (Fabaceae) from past to future were compared through ecological niche modeling (ENM). In the 58 environmental variables used for modeling, ENM, multivariate environmental similarity surfaces and most dissimilar variable analyses showed soil and temperature were the primary factors affecting the distributions. In the past, most species experienced contractions in the last glacial maximum (LGM) while expansions during the Middle Holecene. Distribution range sizes during the LGM in the EAS were much larger. In the future (2050 and 2070), northward movements and range expansions were shared between the EAS and ENA species. Projections of niche of the EAS species to the ENA region resulted in significantly smaller areas in the ENA, while similar areas were found for the vice versa projections. Principal component analysis indicated that ecological niches of the ENA species differed from those of EAS species. Niche identity and background tests showed that most species pairs (10/11) rejected (fully or partially) the null hypothesis of ecological niche equivalence. In conclusion, differences in environmental heterogeneity, historical environmental changes, and niches of *Gleditsia* species between the EAS and ENA contribute to different distribution shift patterns. This study provide additional insights into the biodiversity distribution bias observed in the Northern Hemisphere.

## Graphical abstract

To further understand the mechanism underlying the uneven distribution of biodiversity between Eastern Asia (EAS) and Eastern North America (ENA), we compared the shifts of potential distributions of *Gleditsia* from past to future. Distinct differences were observed in the past, whereas similar shifts were observed in the future. The combination of differences in environmental heterogeneity, historical environmental changes, and niche of *Gleditsia* species contributed to the different shift patterns of distributions in the EAS and ENA. Our findings improve the understanding of biodiversity distribution bias in the Northern Hemisphere.

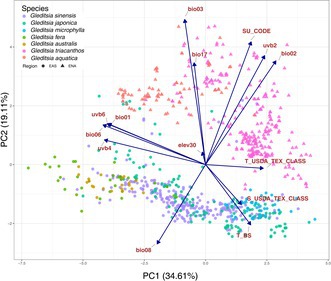

## 1 Background

The spatial and temporal distribution of biodiversity is uneven, guided by several underlying mechanisms (Loreau and De Mazancourt 2013; Schluter and Pennell 2017). Biodiversity anomaly refers to regions with similar habitat while have varied biodiversity (Qian 2002; Qian and Ricklefs 2000). Eastern Asia (EAS) and Eastern North America (ENA) represent anomalies in species diversity with similar land area and latitudinal range, while the EAS flora supports approximately 1.6 times as many species as the ENA flora (Qian and Ricklefs 2000). In the approximately 65 seed plant genera with disjunct distribution in the two regions, higher species diversity is also found in the EAS (Wen 1999). To examine the mechanism behind the species anomaly, regions with similar environments but different diversity patterns must be assessed (Hu et al. 2022).

In the EAS, extreme physiographical heterogeneity, which in conjunction with climate and sea level changes provides abundant opportunities for evolutionary radiation through allopatric speciation, which result in much higher species diversity than the ENA (Qian and Ricklefs 2000). The EAS also provides greater net diversification due to less extinction which is facilitated by more refugia provided by its complex topography (Adams 2009). While the ENA flora experiences much more extinction due to glaciation in its northern regions and aridification in the western regions (Ricklefs and Schluter 1993; Qian and Ricklefs 2000). The greater geological age of the EAS allowed early diversification prior to the subsequent dispersal into the ENA (Ricklefs and Schluter 1993). Moreover, a broad connection between tropical and subtropical flora allows immigration from southeast Asia (Axelrod et al. 1996). Similar immigration is largely lacking in the ENA. Recently, Hu et al. (2022) suggested that higher phylogenetic diversity of angiosperms in the EAS is likely caused by differences in the local orogeny and the associated environment. The substantial anomaly in diversity results from a combination of these factors. However, a key factor explaining the anomaly is likely the presence of a more heterogeneous environment (such as varied topography, temperature, precipitation, and historical climate change) in the EAS.

Species distribution models provides a comparative method for exploring the different responses of the EAS and ENA species to different environmental conditions (Melton et al. 2022; Yin et al. 2021). The maximum entropy model (MaxEnt) shows a higher modeling accuracy than other methods (Phillips et al. 2009), even in the absence of explicit species distribution coordinates. MaxEnt is widely recognized as one of the most effective prediction techniques in ecological research (Li et al. 2021; Liu et al. 2016). For example, Wu et al. (2024) found different distribution shifts from past to present, while similar distribution shifts from present to future in two subregions of subtropical evergreen broadleaved forests (EBLF) in the EAS using Magnoliaceae as a case study through MaxEnt.

*Gleditsia* (Fabaceae) is a small genus with disjunct distribution in the EAS and ENA (Lu et al. 2021). It comprises 13 species that are mainly distributed in the EAS ( *G. australis* , *G. delavayi*, *G. fera*, *G. japonica* , *G. microphylla* , *G. rolfei*, and *G. sinensis* , *G. saxatilis* ), and the ENA ( *G. aquatica* and *G. triacanthos* ) (Lu et al. 2021; Schnabel et al. 2003). *Gleditsia* is likely to have originated in the EAS during the Early to Middle Eocene, and the EAS‐ENA disjunction was formed through migration via the Bering land bridge (Schnabel et al. 2003). *Gleditsia* represents one of the best genera that helps understand the complexities involved in the biogeographic history of the EAS‐ENA disjunctions (Schnabel et al. 2003).

In this study, the potential distributions of *Gleditsia* in the EAS and ENA during the last glacial maximum (LGM), Middle Holocene (MH) in the past, at present, and in 2050 and 2070 in the future were modeled using MaxEnt. The shifts of distributions from past to future were compared to deeper the understand of the underlying mechanism of biodiversity anomaly between the EAS and ENA. The specific questions that this study aimed to answer were: (1) What are the key environmental variables affecting the potential distribution of *Gleditsia* species, and (2) Whether the *Gleditsia* species in the EAS and ENA experience different shift patterns in the future compared to those in the past.

## 2 Methods

### 2.1 Occurrences and Environment Data

**FIGURE 1:**
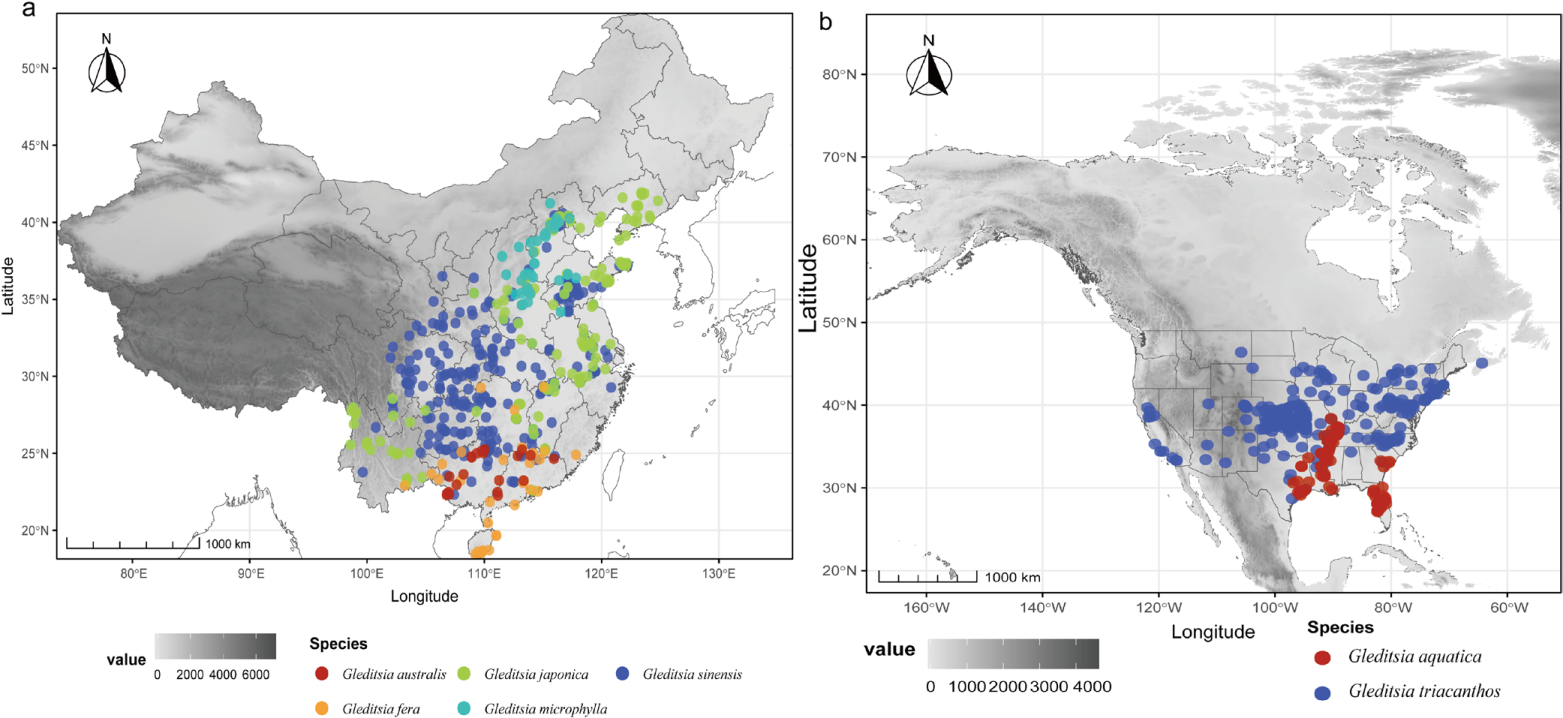
Distribution of occurrence points of *Gleditsia* species in Eastern Asia (a) and Eastern North America (b). Value represents the altitude (m).

The occurrences of native wild *Gleditsia* species (excluding hybrid and cultivated species) were accessed in Chinese Virtual Herbarium (http://www.cvh.ac.cn/), National Specimen Information Infrastructure (http://www.nsii.org.cn/), Germplasm Bank of Wild Species (http://www.genobank.org/), and Global Biodiversity Information Facility (http://www.gbif.org/). Occurrences that were artificially planted or incorrectly recorded were excluded. Duplicate occurrences within 10 km × 10 km were excluded to reduce the bias resulting from the administrative division of the county‐level data. Species with fewer than 20 occurrences were excluded because limited occurrences could lead to biased model simulations (Yin et al. 2021). A total of seven *Gleditsia* species were selected (Figure 1, Table S1, and Figure S2).

We compiled 58 environmental variables, including elevation, 19 climactic factors, 32 soil variables, and six UV‐B variables (Table S3). The climactic variables were downloaded from WorldClim (http://www.worldclim.org/) at a resolution of 30 s (2.5 min in the LGM). The Community Climate System Model 4 (CCSM4) (Shields et al. 2012) during the LGM was selected. In the future, we selected the climate under the most typical concentration trajectory, RCP8.5. The 32 soil variables were obtained from the Harmonized World Soil Database (30 s) (http://www.fao.org/soils‐portal/data‐hub/en/), and UV‐B variables were obtained from glUV (http://www.ufz.de/gluv/) (15 min). All the data were resampled at a spatial resolution of 30 s. The elevation, soil, and UV‐B variables in the past and future were consistent with those used in the present (Wang et al. 2019).

The variables were filtered using multiple steps. At first, all 58 variables at present and default parameters were used for the simulation in MaxEnt 3.3 (Phillips et al. 2006). Then, variables with little contribution percentage < 0.2 were removed (Wu et al. 2024). For two variables whose correlation was > 0.8, or whose variance inflation factor (VIF) was > 5 (Wu et al. 2024), only the variable that exerted higher contribution was retained (Wang et al. 2023). Finally, the remaining variables were used to model the niche of each species. The three variables with the highest contributions were integrated to identify the most important ones.

### 2.2 Model Simulation and Evaluation

MaxEnt was used to simulate potential distributions in the present, past (LGM and MH), and future (2050 and 2070). The background manipulation with bias files for a target group of species method was applied to prevent simulation bias caused by uneven sampling (Phillips et al. 2009). We used 20,000 background points to characterize the available environmental conditions (Phillips et al. 2006, 2009). All background points were randomly selected within 200 km of the ranges of the focal species, with points at the same locations as the presence records excluded (Barbet‐Massin et al. 2012; Barve et al. 2011).

To enhance the transferability of the model, only linear (L) and quadratic (Q) feature functions were selected, and regularization multiplier values of 0.2, 0.4, 0.6, 0.8, 1, 1.5, 2, 2.5, 3, 3.5, 4, 4.5, and 5 were evaluated (Elith et al. 2010). The contribution of each environmental variable was evaluated using jack‐knife testing and a set cross‐validation function with a logistic output. Cross‐validation was applied for species with more than 25 occurrences (Tang et al. 2018). For species with fewer than 25 occurrences ( *G. australis* ), jackknife testing was performed (Pearson et al. 2007).

The receiver operating characteristic (ROC) curve area under the curve (AUC) was used to evaluate performance of modeling (Fawcett 2006). The continuous Boyce index (CBI) was used for models constructed using the cross‐validation method. Leave‐one‐out was used for models constructed using the folding knife method (Bellamy and Altringham 2015). For models using the folding knife, the success rate (q, percentage of correct predictions) and statistical significance were used.

### 2.3 Changes in Distribution Area and Centroids

The maximum training sensitivity plus specificity strategy was used to determine the habitat (presence) of the species (Elith et al. 2010; Li et al. 2019; Liu et al. 2016). The distribution areas of each species were calculated based on the presence of habitats. Changes in range sizes from LGM to present (LGM−present) and from present to future (2070–present) were calculated as (*α*−*β*)/*β*, where “*α*” represents the range size of the species during the LGM or 2070, and “*β*” represents the range size of the species at present.

The species distribution centroids of each species in different periods were analyzed using the SDMTool (Naimi and Araújo 2016) package in R 3.3 (https://www.r‐project.org/), and the shifts of the centroids across the five periods (LGM, MH, present, 2050 and 2070) were illustrated. The SDMToolsbox (Brown 2014) in ArcGIS 10.0 (https://desktop.arcgis.com) was used to calculate the distance of centroid shifts.

### 2.4 Ecological Niche Comparison Between the EAS and ENA

To better understand the differences of *Gleditsia* species in response to climate change between the two regions, we projected the niche of *Gleditsia* species in the two regions onto nonlocal areas separately and then calculated the respective distribution areas. Multivariate environmental similarity surfaces (MESS) (Elith et al. 2010) was used to evaluate environmental dissimilarities between different time periods using the screened variables in the species distribution models. Most dissimilar variable analysis (MoD) (Li et al. 2016) was used to identify the key environmental variables contributing to dissimilarities among different periods.

The 58 environmental variables of all species were subjected to principal component analysis (PCA) using the “rastPCA” function in the “RStoolbox” (Muller et al. 2025) and “ENMTools” packages (Warren et al. 2010). Niche identity and background tests were performed with 1000 pseudorelicated data sets using the R package “ENMTools” . The niche identity test combines the occurrence data for a species pair and then randomly redistributes occurrences to each species with the same size to generate ENM. The background test generat a null distribution for the ENM difference expected between one population and occurrence points placed at random within the range of another population. Niche similarity was quantified using Schoener's *D* and the standardized Hellinger distance (*I*). The hypothesis of niche identity is rejected when the empirically observed value is significantly lower or higher than the values expected from the pseudoreplicated data sets (Warren et al. 2010).

## 3 Results

### 3.1 Model Performance and Variable Contributions

A total of 823 occurrences were collected (Figure 1, Tables S1 and S2, Figures S1 and S2). The mean value of the CBI/q was 0.97, which ranged from 0.95–0.99 (Table S2), and the AUC values were also all > 0.92 (Table S1).

The contribution percentage and jackknife test showed that the key environmental variables influencing the distribution of *Gleditsia* species were soil type (SU_CODE), mean diurnal range (bio02), mean temperature of driest quarter (bio09), and minimum temperature of the coldest month (bio6) (Table S2, Figures S3 and S8).

### 3.2 Shifts of Potential Distributions of *Gleditsia*

At present, in the EAS, *G. japonica* exhibited the widest range, spanning across subtropical and northern China, the Korean Peninsula, and the Japanese Archipelago. *G. microphylla* was primarily found in northern China. *G. australis* and *G. fera* occupied similar distriubtions in the subtropical and tropical regions of China. In the ENA, the most suitable distribution for *G. triacanthos* and *G. aquatica* were in the eastern and southeastern regions, respectively (Figure 1, Figure S1).

**FIGURE 2:**
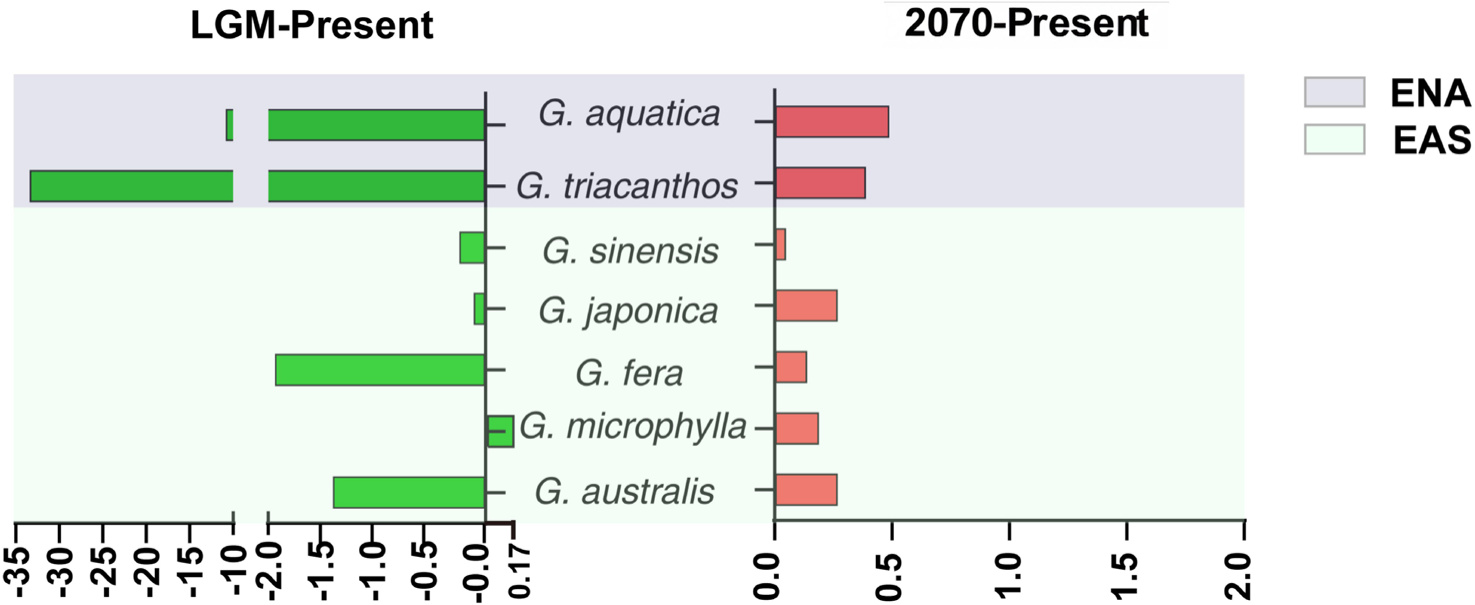
Changes in range size of the potential distributions from last glacial maximum (LGM) to present (LGM‐present, green color), and from present to future at the 2070 (2070‐present, red color) in Eastern Asia (EAS) and Eastern North America (ENA) calculated by (*α* − *β*)/*α*, where “*α*” represents range size of species at the LGM or the 2070, and “*β*” represents the range size of species at present. *G*. in the species names is an abbreviation for the genus *Gleditsia* (e.g., *G. aqustica* refers to *Gleditsia aqustica*).

**FIGURE 3:**
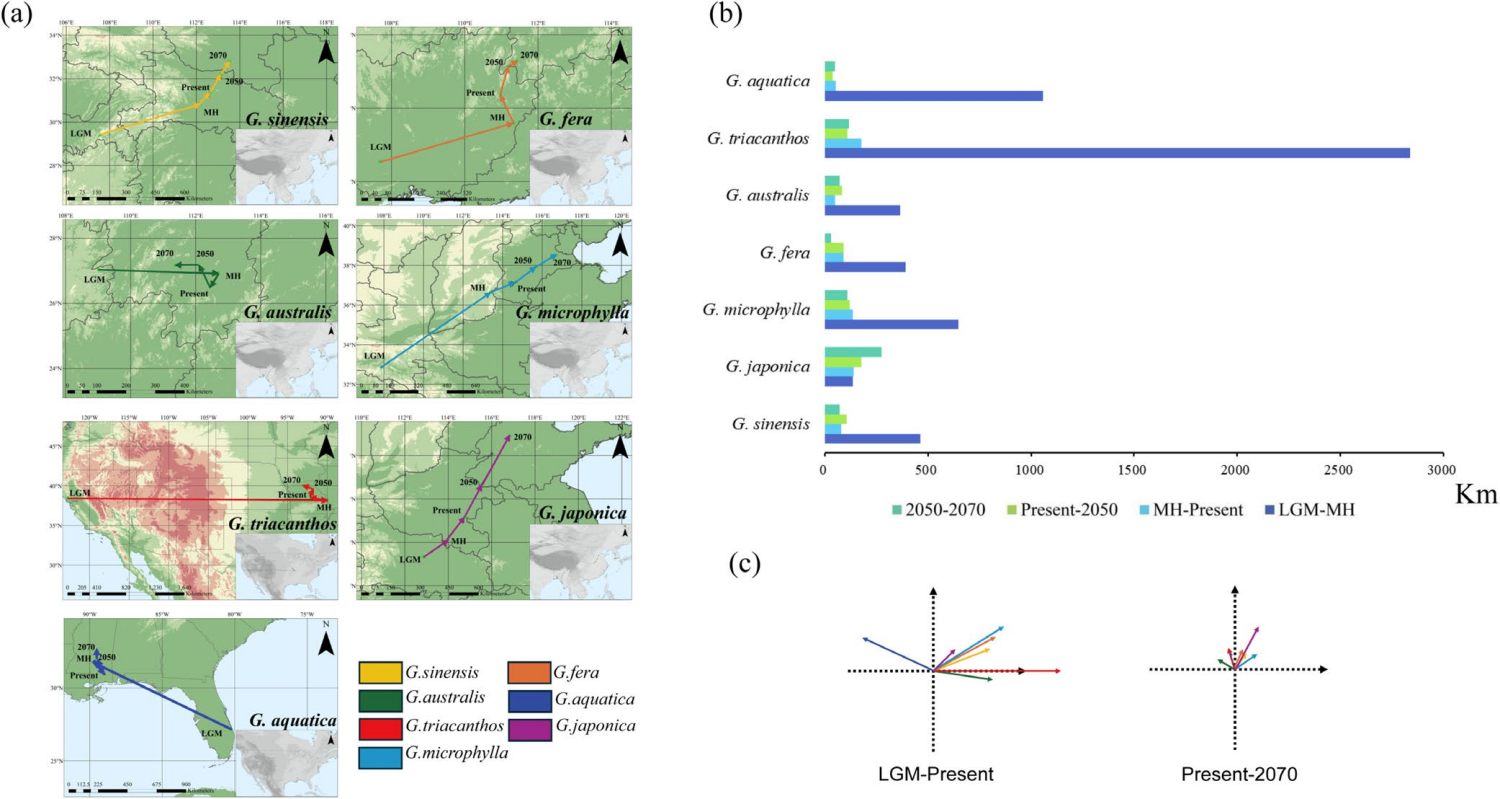
(a) A comparison of the movement of centroids during different periods in Eastern Asia (EAS) and Eastern North America (ENA). (b) Geographical displacement distance of the centroids of potential distributions for *Gleditsia* species during different periods. (c) Summary of the distance and direction of range shifts of the studied species in EAS and ENA from the LGM to present and present to the 2070. *G*. in the species names is an abbreviation for the genus *Gleditsia* (e.g., *G. aqustica* refers to *Gleditsia aqustica*). LGM is an abbreviation for the last glacial maximum.

**FIGURE 4:**
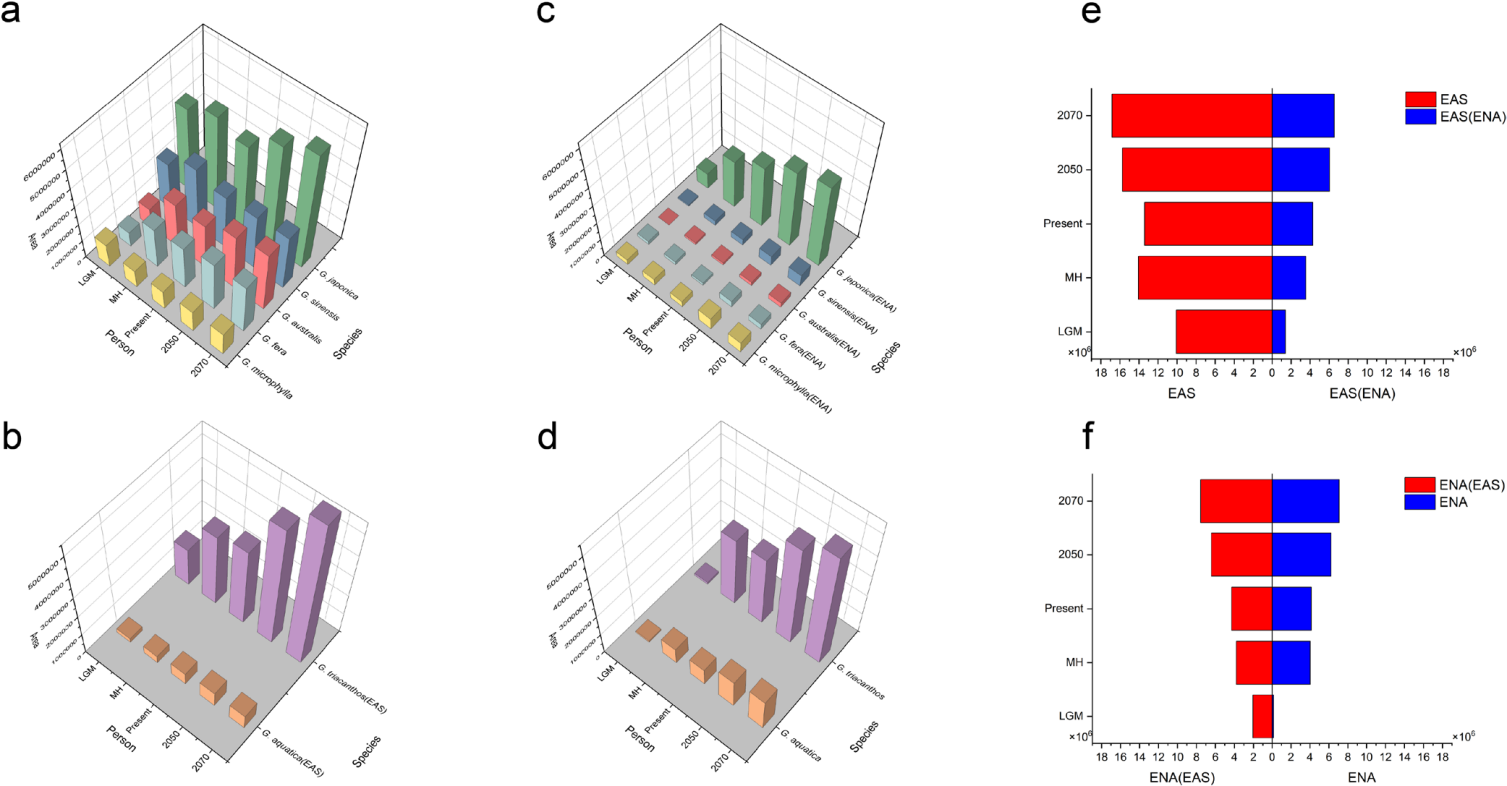
Potential distribution areas of *Gleditsia* species in Eastern Asia (EAS) and Eastern North America (ENA). (a and b) The potential range areas of *Gleditsia* species in their native range. (c and d) The potential range areas of *Gleditsia* species in their non‐native range. (e and f) Comparisons of distribution areas of native and non‐native projections for *Gleditsia* species. *G*. in the species names is an abbreviation for the genus *Gleditsia* (e.g., *G. aqustica* refers to *Gleditsia aqustica*). LGM and MH are abbreviations for the last glacial maximum and Middle Holocene, respectively.

In the past, all *Gleditsia* species had undergone range contractions (except *G. microphylla* ), species in the ENA experience much severer contractions compare to species in the EAS (Figures 2 and 4). In the MH, larger distribution areas were found in all species, except *G. microphylla* (Figure 4a,c). From the LGM to present, the centroids of EAS species mainly moved northeastwards (except *G. australis* ); thus, the habitat gain was mainly located in the higher latitude regions (Figure 3, Figures S1 and S7), whereas in the ENA, the centroids moved northwestwards ( *G. aquatica* ) or eastwards ( *G. triacanthos* ) (Figure 3). In the future, range expansions were found in *Gleditsia* in both EAS and ENA, and range sizes in the 2050 and 2070 were similar (Figures 2 and 3, Figure 4a,c). Northward or northeastward migrations of the centroid were observed in almost all species, except *G. australis* , which shifted its centroid northwestward (Figure 3).

### 3.3 Niche Comparison of *Gleditsia* Between the EAS and ENA

The areas of potential distributions of *Gleditsia* species in the EAS were significantly reduced when projected onto the non‐native (ENA) region across all periods (Figure 4, Figures S1 and S4). Similar distribution areas were found for ENA species in the projection to non‐native (EAS) region across all periods, except for the LGM that showed a significantly larger distributions in the EAS compared to that in the ENA (Figure 4b,d, Figures S1 and S4).

**FIGURE 5:**
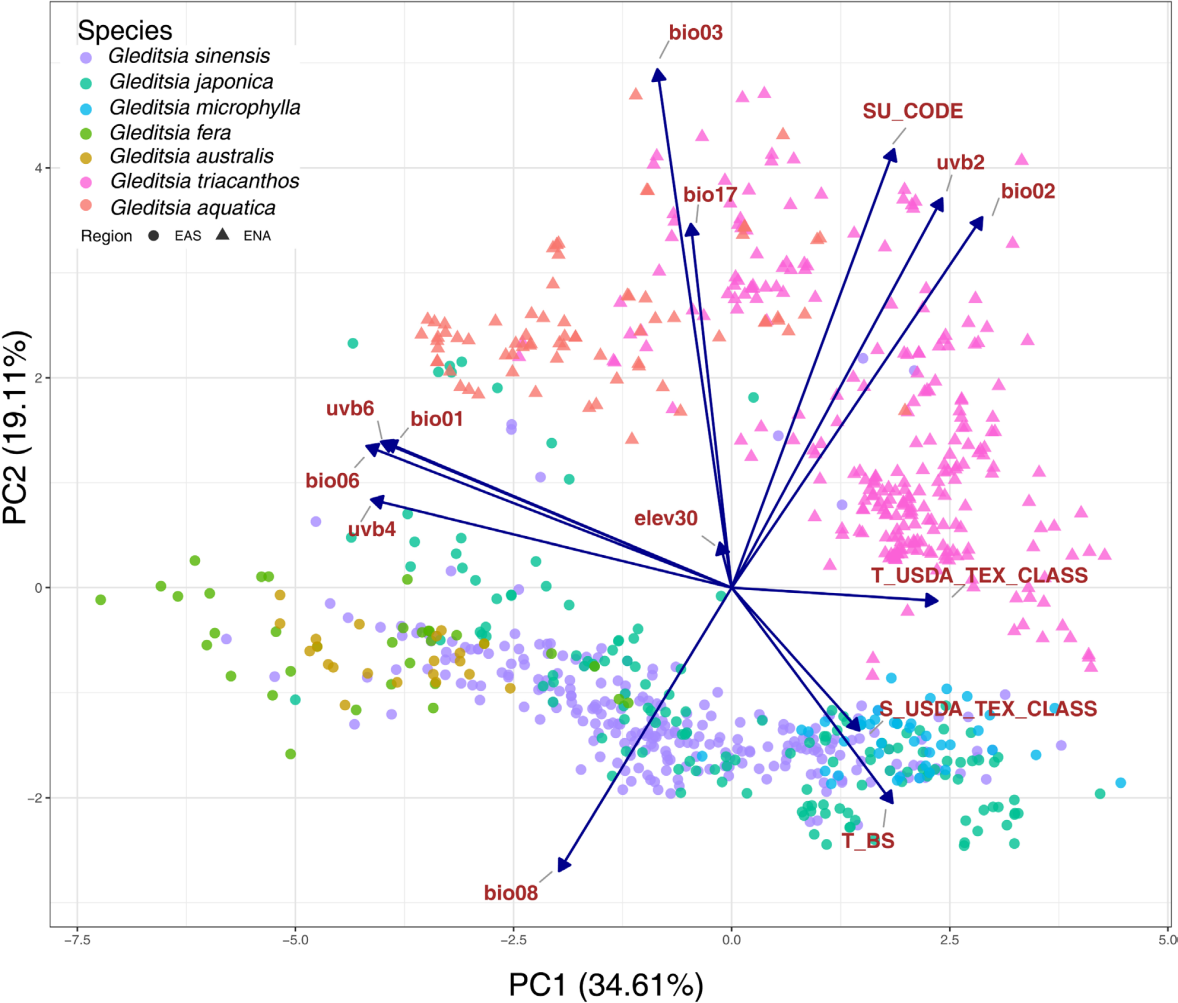
Principal component analysis (PCA) plots based on environmental variables at *Gleditsia* occurrence points. Different shapes correspond to the Eastern Asia (EAS) and Eastern North America (ENA) regions.

In the PCA analysis, the first two axes explained 53.72% (PC 1:34.61%, PC 2:19.11%, Figure 5) of the total variations; PC1 and PC2 were mainly associated with temperature (bio06, bio01, bio03 and bio02), UV‐B‐ (uvb4, uvb6 and uvb2), and soil (SU_CODE) variables (Figure S5). We found that *G. microphylla* had the smallest ecological niche, whereas *G. sinensis* and *G. japonica* had the largest. The ecological niches of the ENA species differed from those of the EAS species (Figure 5, Figures S3 and S5). Full (both *D* and *I* index) rejections to the null hypotheses were found in majority species pairs in niche identity tests (7/11) and background test (7/11). Partial (either *D* or *I* index) rejections were found in three species pairs, *G. sinensis* vs. *G. japonica, G. sinensis* vs. *G. australis*, and *G. japonica* vs. *G. australis*, in niche identity or background tests. Acceptance to the null hypotheses was found in species pairs of *G. australis* vs *G. fera* (Tables S4 and S5, Figures S9 and S10).

MESS analysis revealed that the environment in the EAS was relatively stable and while unstable in the ENA across different periods (Figures S6 and S11). The MoD analysis indicated that soil‐ and temperature‐related factors (such as SU_CODE, bio01, bio02, bio06, bio08, and bio09) contributed to the dissimilarities between the different periods (Figure S11).

## 4 Discussion

### 4.1 Key Variables Affecting the Potential Distributions of *Gleditsia* Species

In this study, changes in the potential distributions of *Gleditsia* from past to future were compared to better understand the mechanism affecting the biased distribution of biodiversity between the EAS and ENA. Different analyses (ENM, MoD, and PCA) revealed that soil and temperature were likely the primary factors affecting the distribution of *Gleditsia* among approximately 60 different environmental variables (Figure 5, Figures S1 and S11).

Soil is an important variable that varies with longitude, latitude, and elevation (Melton et al. 2022). However, it is seldom included in niche modeling (Ni and Vellend 2024; Wang and Guan 2023). Ni and Vellend (2024) compared the migrations of 1870 species and reported that models employ soil variables predict much smaller northward migrations in the ENA compared to models that use only climate variables. In the EAS, soil also contributes significantly to the potential distribution modeling of Magnoliaceae from the past to the future (Wu et al. 2024). Other taxa‐specific studies have also shown the importance of soil in predicting species distribution (Ray et al. 2016; Wang and Guan 2023).

Temperature and precipitation, or either of them, are the main factors influencing the distribution of plants (Tang et al. 2018). In the EAS‐ENA disjunct genus *Cornus*, temperature and precipitation, combined with elevation, are the main contributing variables for potential distributions (Lindelof et al. 2020). In *Chamaecyparis*, greatly reduced precipitation and temperature during the LGM in the Northern Hemisphere might have greatly impacted its distributions (Liu et al. 2017). Similarities can be found in the EAS, distribution of species in some dominant family, such as Lauraceae (Liao et al. 2022), Fagaceae (Hai et al. 2022), and Theaceae (Tang and Zhao 2022), are affected by both temperature and precipitation variables, while in Magnoliaceae, temperature is the main affecting variables (Wu et al. 2024), similar to that of *Gleditsia* species. Precipitation could also exert great influence. In the EAS‐ENA disjunct distributed *Nyssa*, different precipitation variables (bio14, bio17, bio19, and bio15) show correlations to 14 different morphological characters, and in EAS and ENA disjunct sister clade ( *N. sinensis* and *N. sylvatica‐N. biflora*), precipitation variables (bio13 and bio16) also show differentiation (Zhou et al. 2020). However, the precipitation variables have very little contribution in distribution of *Gleditsia* species (Figure S3), indicating the variables that are responsible for distributions of EAS‐ENA disjunct distributed genera varies among different genera.

### 4.2 Comparison of Distribution Shifts of *Gleditsia* Between the EAS and ENA

During the LGM, range shrinkage was observed for all *Gleditsia* species (except *G. microphylla* ), which is consistent with previous paleo‐vegetation constructions (Harrison et al. 2001; Ni et al. 2010). However, the range contraction (to the westernmost or southernmost region) was much more severe in the ENA than that in the EAS, as the environment experienced substantial changes in the ENA (Figure S7). *Gleditsia* species can survive in much larger and wider refugia, such as the Hengduan Mountains (Liang et al. 2018), subtropical regions (Ye et al. 2017), north China (Qiu et al. 2011), Korea Peninsular and Japanese Archipelago (Qiu et al. 2011; Ye et al. 2017) in the EAS, especially in the mountains subtropical regions, in situ suivival is realized by altitude changes rather than latitude changes in response to climate fluctuations during glacial–interglacial changes in the Quaternary. A much more stable environment from the LGM to the present (Figure S6) provides further evidence. The occurrence of less ice coverage in the EAS than in the ENA also contributes to this pattern, resulting high genetic diversity in the EAS (Qiu et al. 2011). Substantially broader distributions of projecting the niche of *Gleditsia* species from ENA to EAS are also caused by highly heterogeneitous environment in the EAS. In the MH, northeastward shift and range area observed for all *Gleditsia* species (except *G. microphylla* ). Differential shift patterns in *G. microphylla* are likely because it distributes in the transitional region of subtropical and temperate region (Ni et al. 2010), and it has the smallest ecological niche range (Figure 5).

At present, niche identity and background tests indicate niche divergence is dominant in *Gleditsia* species. While similarities are found more often in the EAS species than that in the ENA species, especially in *G. australis* and *G. fera*. Different flowering phase (June to October in *G. australis* and April to May in *G. fera*, Flora of China, https://www.iplant.cn/) may responsible for speciation of the two species with sympatric distribution in tropical‐subtropical region. Apart from divergence in flowering phase, mountains uplift, monsoon intensification, sea level changes and other historical changes in conjunction with extreme physiographical heterogeneity, provide ample opportunities for speciation (Qian and Ricklefs 2000; Hu et al. 2022), resulting in greater species diversity (five species in the EAS compared to two in the ENA).

In the future, potential distribution predictions revealed that northward migration and range expansion will be observed in all *Gleditsia* species in both EAS and ENA region. Global warming has significantly affected species distributions, causing expansions, shifts, or contractions in habitats (Wei et al. 2018). Some species face threats from climate change and have become endangered (Song et al. 2021), whereas others expand their distributions (Liu et al. 2021). Migration to higher latitudes is common under future climate warming, resulting in extended northern range limit of various tree species, resulting in range expansion (Hamann and Wang 2006). The slightly higher rate of expansion in the ENA than in the EAS may be attributed to two reasons. First, less environmental heterogeneity in the high‐latitude regions than in the southern mountainous regions is likely to cause a range decrease in most species in the EAS, with only a few species experiencing range expansion (Wu et al. 2024). Second, much greater dissimilarity in the environment between the future and the present in the ENA may provide a more suitable habitat for the survival of *Gleditsia* species.

In the EAS, extreme physiographical heterogeneity, climate and sea‐level changes (Qian and Ricklefs 2000), greater net diversification related to complex topography (Adams 2009), greater geological age (Ricklefs and Schluter 1993), broader connection between tropical and subtropical flora (Axelrod et al. 1996), differences in the local orogeny and the associated environment (Hu et al. 2022) all contributed to the higher biodiversity compare to the ENA. The present study about the ENM of *Gleditsia* species further emphasize the importance of more heterogeneous environment in the EAS (such as varied topography, temperature, precipitation, and historical climate change) (Qian and Ricklefs 2000).

## 5 Conclusions

To further understand the mechanism underlying the uneven distribution of biodiversity between the EAS and ENA, we compared the shifts of potential distributions of *Gleditsia* from the past to the future in the two regions. Distinct differences were observed in the past, whereas similar shifts were observed in the future. The combination of differences in environmental heterogeneity, historical environmental changes, and the niche of *Gleditsia* species contributed to the different shift patterns of distributions in the EAS and ENA. Our findings deeper the understanding of biodiversity distribution bias in the Northern Hemisphere.

## Author Contributions

**Zhao‐Yu Yan:** data curation (lead), formal analysis (lead), project administration (lead), validation (equal), visualization (lead), writing – original draft (lead), writing – review and editing (equal). **Hai‐Yang Wu:** methodology (lead). **Bin Tian:** conceptualization (equal), funding acquisition (equal), resources (equal), supervision (equal), writing – review and editing (equal). **Jun‐Wei Ye:** conceptualization (equal), funding acquisition (equal), resources (equal), supervision (equal), visualization (equal), writing – original draft (equal), writing – review and editing (equal).

## Funding

This work was supported by Natural Science Foundation of China (NSFC, 32260308, 32260056), Ten thousand Talents Program of Yunnan Province (YNWR‐QNBJ‐2020‐232, YNWR‐QNBJ‐2020‐292), Xingdian Talent Support Program (XDRC‐QNRC‐2022‐0323), and Yunnan Fundamental Research Projects (202301AU070224). We thank all the individuals who helped us in this study.

## Conflicts of Interest

The authors declare no conflicts of interest.

## Supporting information


**Data S1:** ece372591‐sup‐0001‐DataS1.xlsx.

**Appendix S1:** ece372591‐sup‐0002‐Supinfo.docx.

## Data Availability

All the required data are uploaded as Supporting Information.
