# Supplementary material for: Comparison of Shifts of Potential Distributions in Gleditsia (Fabaceae) Between Eastern Asia and Eastern North America
Source: Ecol Evol. 2025 Nov 29;15(12):e72591. doi: 10.1002/ece3.72591 (PMC12664105; doi:10.1002/ece3.72591)
Supplement: Supplementary file 2 — Appendix S1: ece372591‐sup‐0002‐Supinfo.docx. [file ECE3-15-e72591-s002.docx]

**Supplementary Material**

Comparison of shifts of potential distributions in *Gleditsia* (Fabaceae) between Eastern Asia and Eastern North America

Zhao-Yu Yan, Hai-Yang, Wu, Bin Tian and Jun-Wei Ye

| **Table S1: The area under the receiver operating characteristic curve (ROC) scores for species distribution models (SDMs) by the following algorithms: the principle of maximum entropy (Maxent). ENA (or EAS) represents the projection of niche of EAS, Eastern Asia, (or ENA, Eastern North America) species into ENA (or EAS) region.** | |
| --- | --- |
| Species | Evaluation metric (AUC) |
| *Gleditsia australis* | 0.97 |
| *Gleditsia fera* | 0.97 |
| *Gleditsia japonica* | 0.93 |
| *Gleditsia microphylla* | 0.99 |
| *Gleditsia sinensis* | 0.94 |
| *Gleditsia tracanthos* | 0.92 |
| *Gleditsia aquatica* | 0.98 |
| *Gleditsia australis (ENA)* | 0.97 |
| *Gleditsia fera (ENA)* | 0.99 |
| *Gleditsia japonica (ENA)* | 0.93 |
| *Gleditsia microphylla (ENA)* | 0.99 |
| *Gleditsia sinensis (ENA)* | 0.94 |
| *Gleditsia tracanthos (EAS)* | 0.92 |
| *Gleditsia aquatica (EAS)* | 0.96 |

| **Table S2: The model and parameters generated in this study and the evaluation results of the model.** | | | | | | | | |
| --- | --- | --- | --- | --- | --- | --- | --- | --- |
| **Species** | **Species presence data** | **Reserved variable** | **Regularized** | **CBI/q** | **MSS** | **A** | **B** | **C** |
| *G. australis* | 21 | 36,37,52,21,2,8,3,18,15,57 | 6 | 0.955 | 0.2153 | 2 | 36 | 37 |
| *G.. fera* | 33 | 37,52,36,21,2,3,1,13,18,45,27 | 3.5 | 0.946 | 0.1352 | 9 | 1 | 37 |
| *G. japonica* | 146 | 37,21,36,52,9,2,15,20,29,39,24,54,34,8,22,13 | 3 | 0.983 | 0.1320 | 9 | 2 | 20 |
| *G. microphylla* | 40 | 37,36,21,52,9,38,15,54,2,22,29,47,24 | 4 | 0.970 | 0.0963 | 9 | 38 | 54 |
| *G. sinensis* | 258 | 37,21,36,6,2,58,57,15,31,29 | 0.2 | 0.993 | 0.1556 | 6 | 37 | 2 |
| *G. tracanthos* | 253 | 37,21,36,52,6,8,56,24,20,5,18,15,22,30,39 | 1 | 0.992 | 0.1810 | 6 | 37 | 8 |
| *G. aquatica* | 72 | 36,37,9,1,17,39,12,27,23 | 6 | 0.955 | 0.2153 | 9 | 1 | 37 |

Retain the variable sequence number correspondence table S3. The abbreviations CBI and *q* represent the Continuous Boyce Index (CBI) and the success rate (the percentage of correct predictions), respectively. MSS refers to the maximum training sensitivity plus specificity strategy. Variables A, B, and C represent the order of variable importance, from highest to lowest, respectively.

| **Table S3:** **The 58 bioclimatic variables that were used for niche modeling.** | | |
| --- | --- | --- |
| **Number** | **Abbreviation** | **Variable Definition** |
| 1 | bio01 | Annual Mean Temperature |
| 2 | bio02 | (Mean of monthly (max temp - min temp)) |
| 3 | bio03 | Isothermality (BIO2/BIO7) (×100) |
| 4 | bio04 | Temperature Seasonality (standard deviation ×100) |
| 5 | bio05 | Max Temperature of Warmest Month |
| 6 | bio06 | Min Temperature of Coldest Month |
| 7 | bio07 | Temperature Annual Range (BIO5-BIO6)) |
| 8 | bio08 | Mean Temperature of Wettest Quarter |
| 9 | bio09 | Mean Temperature of Driest Quarter |
| 10 | bio10 | Mean Temperature of Warmest Quarter |
| 11 | bio11 | Mean Temperature of Coldest Quarter |
| 12 | bio12 | Annual Precipitation |
| 13 | bio13 | Precipitation of Wettest Month |
| 14 | bio14 | Precipitation of Driest Month |
| 15 | bio15 | Precipitation Seasonality (Coefficient of Variation) |
| 16 | bio16 | Precipitation of Wettest Quarter |
| 17 | bio17 | Precipitation of Driest Quarter |
| 18 | bio18 | Precipitation of Warmest Quarter |
| 19 | bio19 | Precipitation of Coldest Quarter |
| 20 | elev30 | Elevation |
| 21 | IAWC_CLASS | Available water storage capacity in mm/m of the soil unit |
| 22 | S_BS | Subsoil Base Saturation (%) |
| 23 | S_CACO3 | the calcium carbonate content in the subsoil |
| 24 | S_CASO4_10 | Subsoil Gypsum (% weight) |
| 25 | S_CEC_CLAY | Subsoil CEC (clay) (cmol/kg) |
| 26 | S_CEC_SOIL | Subsoil CEC (soil) (cmol/kg) |
| 27 | S_CLAY | Subsoil Clay Fraction (% wt.) |
| 28 | S_ECE_10 | Subsoil Salinity (ECe) (dS/m) |
| 29 | S_ESP | Subsoil Sodicity (ESP) (%) |
| 30 | S_OC_100 | the organic carbon in the subsoil |
| 31 | S_PH_H2O | the soil reaction of subsoil |
| 32 | S_REF_BULK_DENSITY_100 | Subsoil Reference Bulk Density (kg/dm3.) |
| 33 | S_SAND | Subsoil Sand Fraction (% wt.) |
| 34 | S_SILT | R Subsoil Silt Fraction (% wt.) |
| 35 | S_TEB | Subsoil TEB (cmol/kg) |
| 36 | S_USDA_TEX_CLASS | Subsoil USDA Texture Classification (name) |
| 37 | SU_CODE90 | Soil classification |
| 38 | T_BS | Topsoil Base Saturation (%) |
| 39 | T_CACO3 | Topsoil Calcium Carbonate |
| 40 | T_CASO4_10 | Topsoil Gypsum (% weight) |
| 41 | T_CEC_CLAY | Topsoil CEC (clay) (cmol/kg) |
| 42 | T_CEC_SOIL | Topsoil CEC (soil) (cmol/kg) |
| 43 | T_CLAY | Topsoil Clay Fraction (% wt.) |
| 44 | T_ECE | Topsoil Salinity (Elco) (dS/m) |
| 45 | T_ESP | Topsoil Sodicity (ESP) (%) |
| 46 | T_OC_100 | the organic carbon in topsoil |
| 47 | T_PH_H2O | the soil reaction of topsoil |
| 48 | T_REF_BULK_DENSITY_100 | Topsoil Reference Bulk Density (kg/dm3.) |
| 49 | T_SAND | Topsoil Sand Fraction (% wt.) |
| 50 | T_SILT | Topsoil Silt Fraction (% wt.) |
| 51 | T_TEB | Topsoil TEB (cmol/kg) |
| 52 | T_USDA_TEX_CLASS | Topsoil USDA Texture Classification (name) |
| 53 | uvb1 | Annual Mean Ultraviolet-B (UV-B) |
| 54 | uvb2 | UV-B Seasonality |
| 55 | uvb3 | Mean UV-B of Highest Month |
| 56 | uvb4 | Mean UV-B of Lowest Month |
| 57 | uvb5 | Sum of Monthly Mean UV-B during Highest Quarter |
| 58 | uvb6 | The sum of Monthly Mean UV-B during Lowest Quarter |

| **Table S4: Sampling for identity testing. D represents Schoener's D, and I represents Hellinger distance (I). EAS stands for East Asia, and ENA stands for the eastern part of North America.** | | | | |
| --- | --- | --- | --- | --- |
| **Species vs Species** | ***D*** | ***p*-value** | ***I*** | ***p*-value** |
| (EAS)*G. sinensis vs G. japonica* | 0.36017 | ***p*** < 0.05 | 0.66164 | ***p*** > 0.05 |
| (EAS)*G. sinensis vs G. microphylla* | 0.17426 | ***p*** < 0.05 | 0.41215 | ***p*** < 0.05 |
| (EAS)*G. sinensis vs G. fera* | 0.19585 | ***p*** < 0.05 | 0.46147 | ***p*** < 0.05 |
| (EAS)*G. sinensis vs G.australis* | 0.37687 | ***p*** > 0.05 | 0.67821 | ***p*** < 0.05 |
| (EAS)*G. japonica vs G. microphylla* | 0.27074 | ***p*** < 0.05 | 0.54160 | ***p*** < 0.05 |
| (EAS)*G. japonica vs G. fera* | 0.22984 | ***p*** < 0.05 | 0.49178 | ***p*** < 0.05 |
| (EAS)*G. japonica vs G.australis* | 0.27819 | ***p*** > 0.05 | 0.53872 | ***p*** < 0.05 |
| (EAS)*G.australis vs G. microphylla* | 0.36136 | ***p*** < 0.05 | 0.65631 | ***p*** < 0.05 |
| (EAS)*G.australis vs G. fera* | 0.55825 | ***p*** > 0.05 | 0.83101 | ***p*** > 0.05 |
| (EAS)*G. microphylla vs G. fera* | 0.11157 | ***p*** < 0.05 | 0.33900 | ***p*** < 0.05 |
| (ENA)*G. aquatica vs G. triacanthos* | 0.35365 | ***p*** < 0.05 | 0.60164 | ***p*** < 0.05 |

**ENA (or EAS) represents the projection of niche of EAS, Eastern Asia, (or ENA, Eastern North America) species into ENA (or EAS) region.**

| **Table S5: Sampling for background test. D represents Schoener's D, and I represents Hellinger distance (I). EAS stands for East Asia, and ENA stands for the eastern part of North America.** | | | | |
| --- | --- | --- | --- | --- |
| **Species vs Species** | ***D*** | ***p*-value** | ***I*** | ***p*-value** |
| (EAS)*G. sinensis vs G. japonica* | 0.4185 | ***p*** < 0.05 | 0.70826 | ***p*** > 0.05 |
| (EAS)*G. sinensis vs G. microphylla* | 0.26393 | ***p*** < 0.05 | 0.54446 | ***p*** < 0.05 |
| (EAS)*G. sinensis vs G. fera* | 0.16196 | ***p*** < 0.05 | 0.38125 | ***p*** < 0.05 |
| (EAS)*G. sinensis vs G.australis* | 0.22717 | ***p*** > 0.05 | 0.52574 | ***p*** < 0.05 |
| (EAS)*G. japonica vs G. microphylla* | 0.23224 | ***p*** < 0.05 | 0.50446 | ***p*** < 0.05 |
| (EAS)*G. japonica vs G. fera* | 0.2417 | ***p*** < 0.05 | 0.47827 | ***p*** < 0.05 |
| (EAS)*G. japonica vs G.australis* | 0.27875 | ***p*** > 0.05 | 0.5452 | ***p*** < 0.05 |
| (EAS)*G.australis vs G. microphylla* | 0.14171 | ***p*** < 0.05 | 0.31803 | ***p*** < 0.05 |
| (EAS)*G.australis vs G. fera* | 0.36345 | ***p*** > 0.05 | 0.6819 | ***p*** > 0.05 |
| (EAS)*G. microphylla vs G. fera* | 0.05689 | ***p*** < 0.05 | 0.18641 | ***p*** < 0.05 |
| (ENA)*G. aquatica vs G. triacanthos* | 0.202 | ***p*** < 0.05 | 0.45065 | ***p*** < 0.05 |

**ENA (or EAS) represents the projection of niche of EAS, Eastern Asia, (or ENA, Eastern North America) species into ENA (or EAS) region.**

**
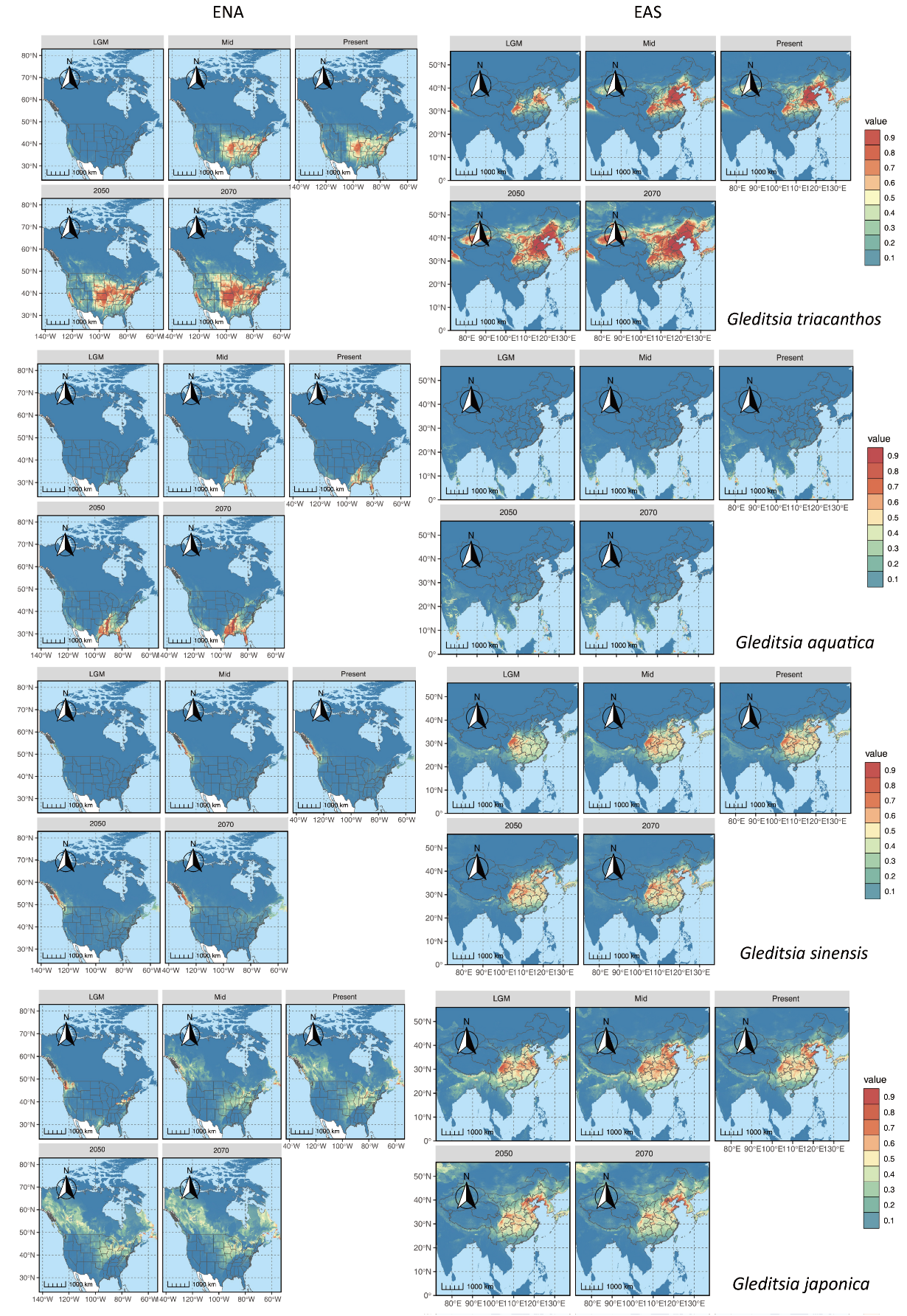
**


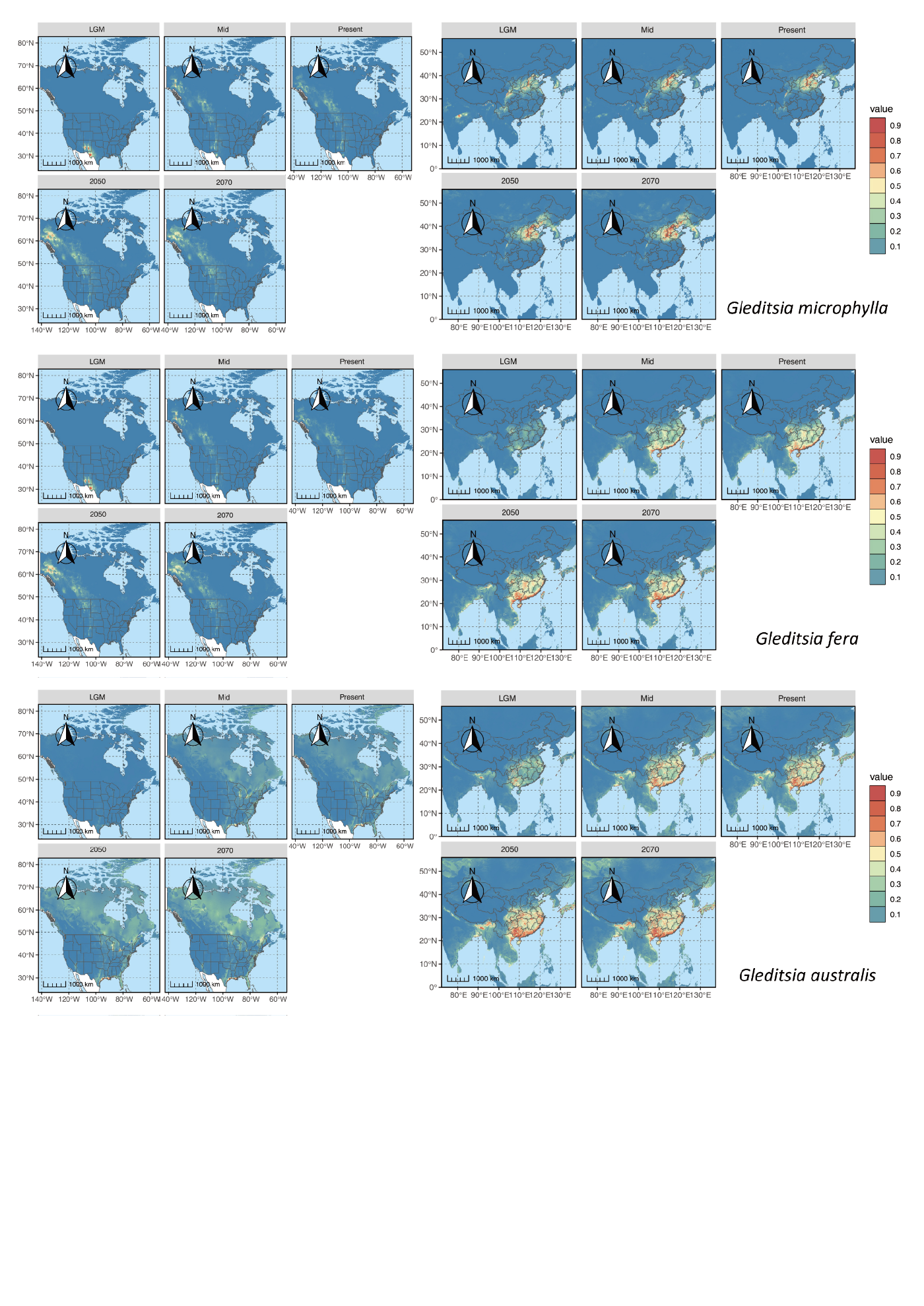


**Figure S1:** Potential distribution of *Gleditsia* species in the ENA and EAS under past, present and future scenarios based on different years using the MaxEnt model. EAS and ENA stand for East Asia and Eastern North America, respectively. LGM and Mid stand for the Last Glacial Maximum and the Mid-Holocene, respectively.


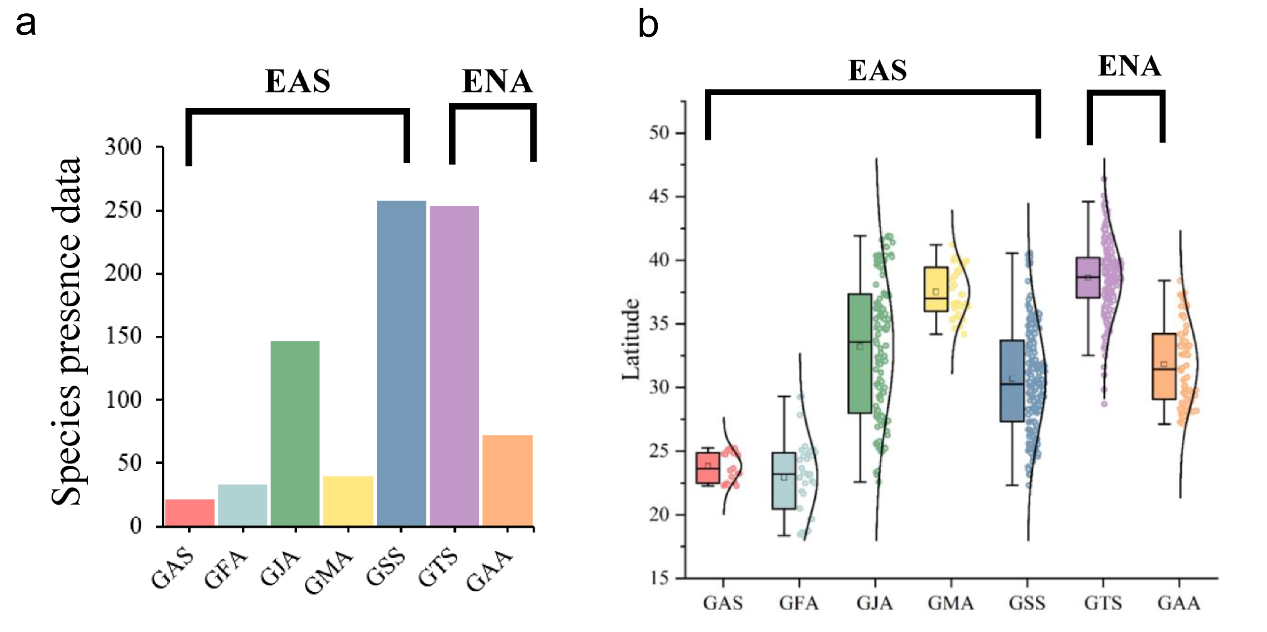


**Figure S2:** The number of distribution points for each species and the latitudinal extent of its distribution. EAS and ENA stand for East Asia and Eastern North America, respectively. GAS: *Gleditsia australis*; GFA: *Gleditsia fera*; GJA: *Gleditsia japonica*; GMA: *Gleditsia microphylla*, GSS: *Gleditsia sinensis*; GTS: *Gleditsia triacanthos*; GAA: *Gleditsia aquatica*.


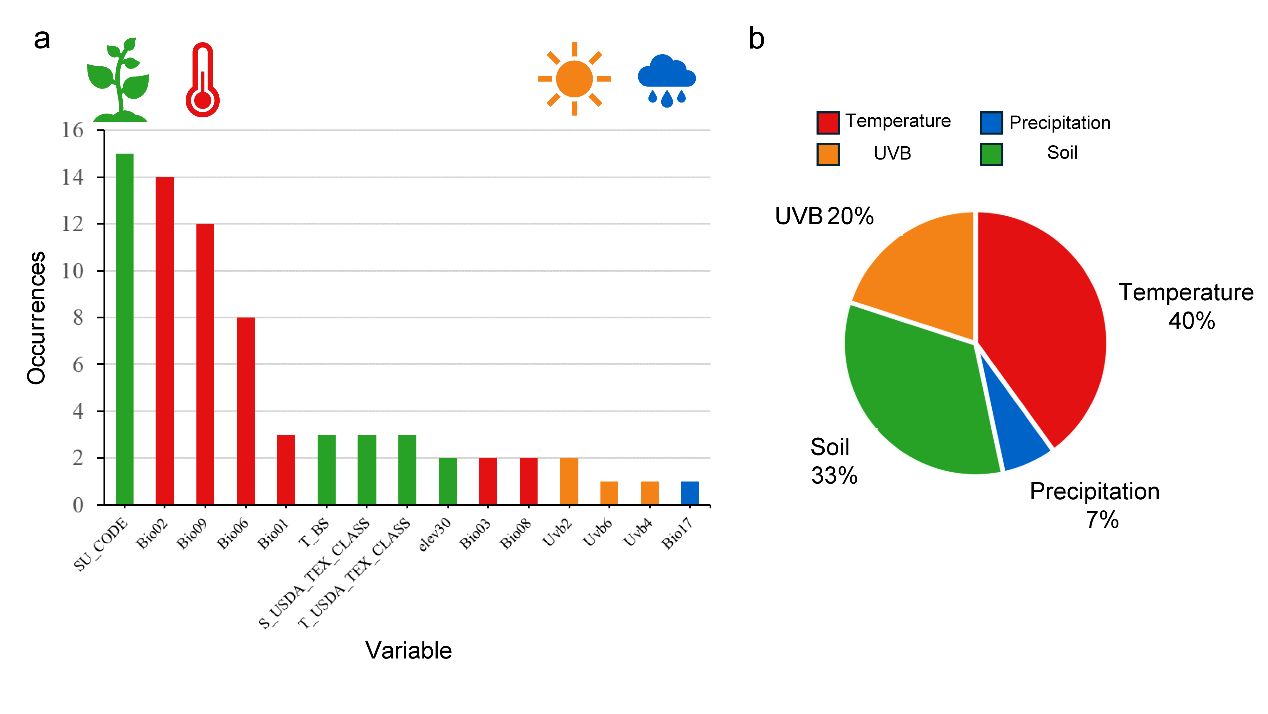


**Figure S3:** The variables appearing in the figure are those with the top three impact contribution rates in the MaxEnt simulation of the *Gleditsia.*


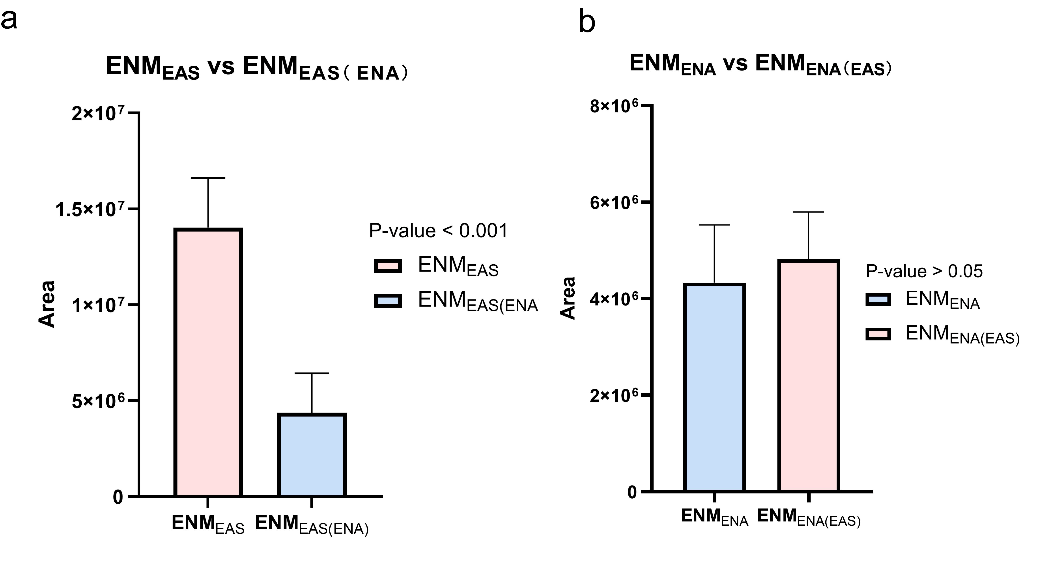


**Figure S4:** The niche area of *Gleditsia* sinensis species was compared between the native projection area and the non-native projection area, with ENM_EAS_ representing the niche area of *Gleditsia* species in EAS area. ENM_EAS(ENA)_ represented the niche area of non-native *Gleditsia* species projected onto ENA in EAS. ENM_ENA_ represents the niche area of *Gleditsia* species in ENA. ENM_ENA(EAS)_ represents the niche area of the species of the genus *Gleditsia* in ENA that is not native projected onto EAS.


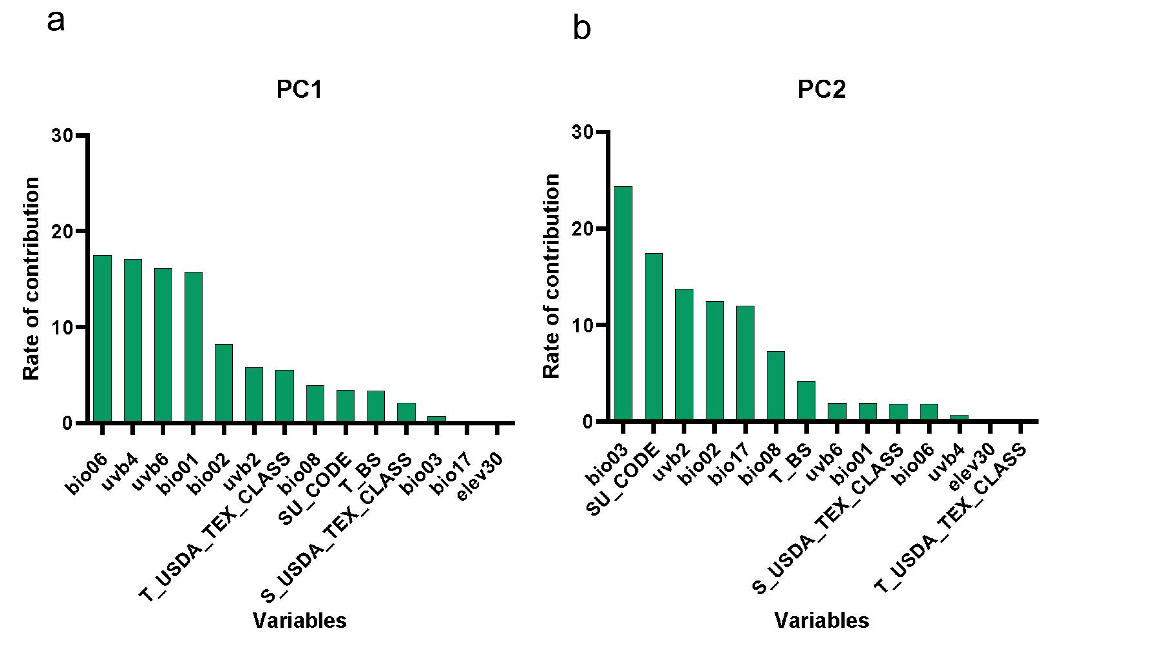


**Figure S5:** Contribution rate of each variable to PC1 axis and PC2 axis.


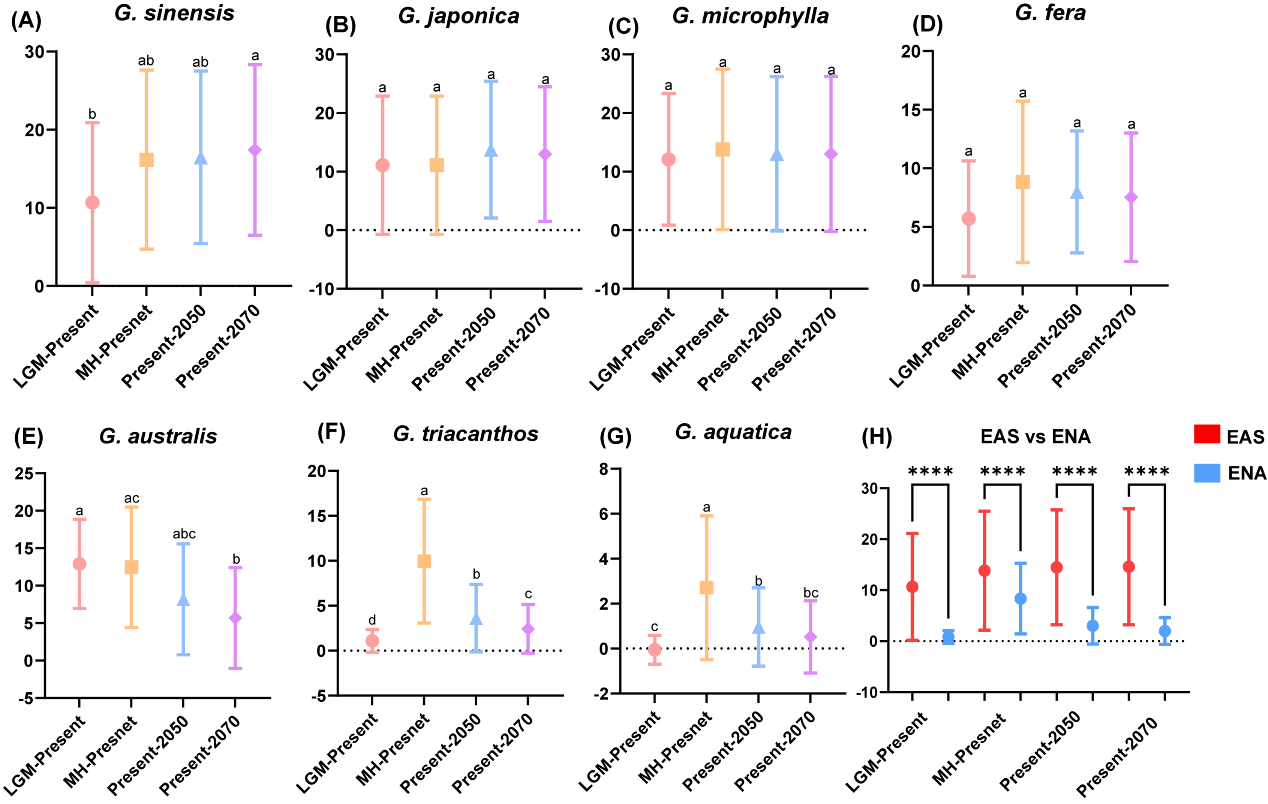


**Figure S6:** Multivariate environmental similarity surface analysis was used to extract values for all species points. Where (A)-(G), light red represents the last glacial maximum to the present (LGM-Present); Orange represents the middle Holocene to the present (MH-Present); Light blue represents the current period to 2050; Purple represents the current period to 2070. (H) represents the comparison between EAS and ENA species of the genus *Gleditsia.* LGM and MH stand for the Last Glacial Maximum and the Mid-Holocene, respectively*.*


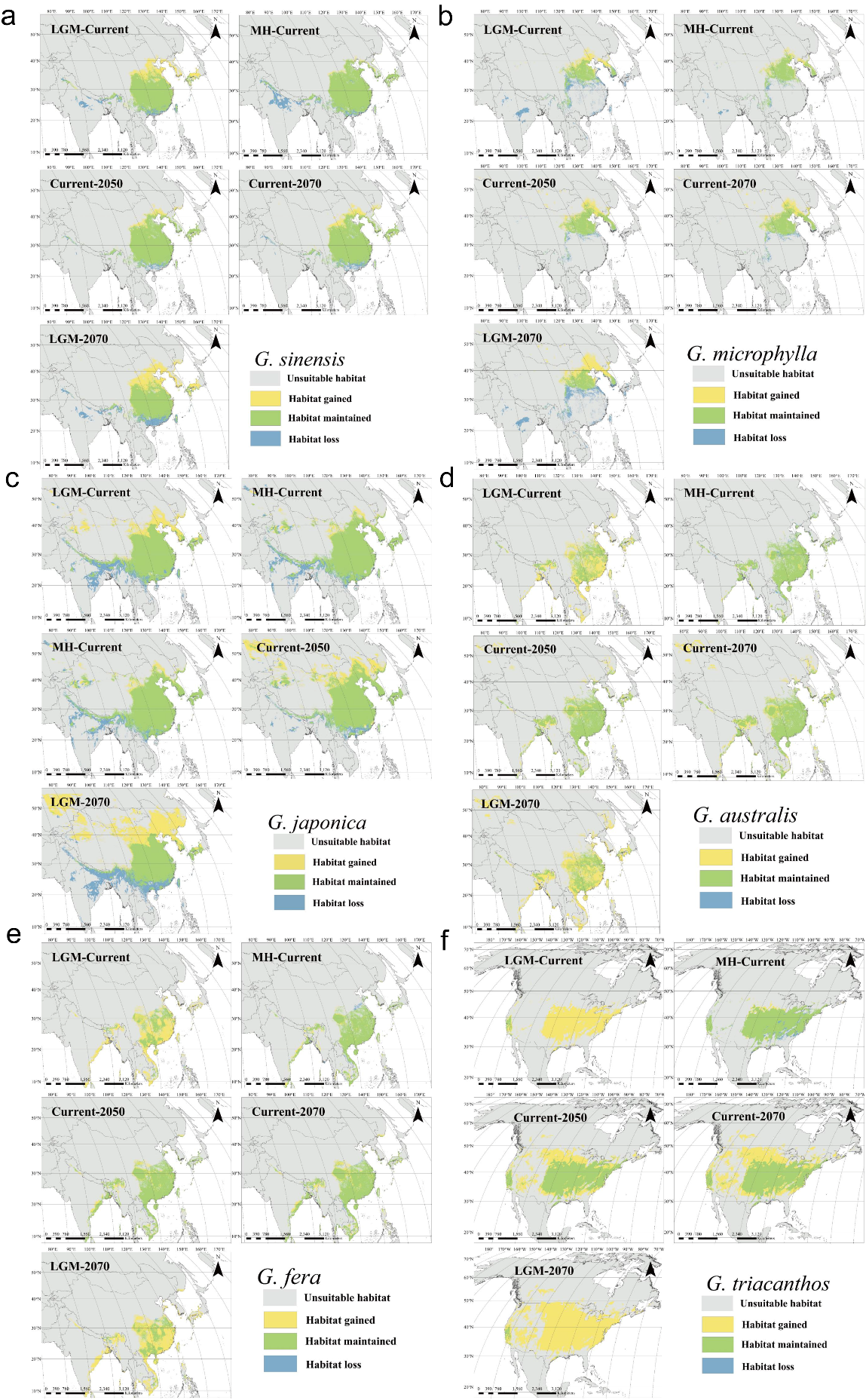


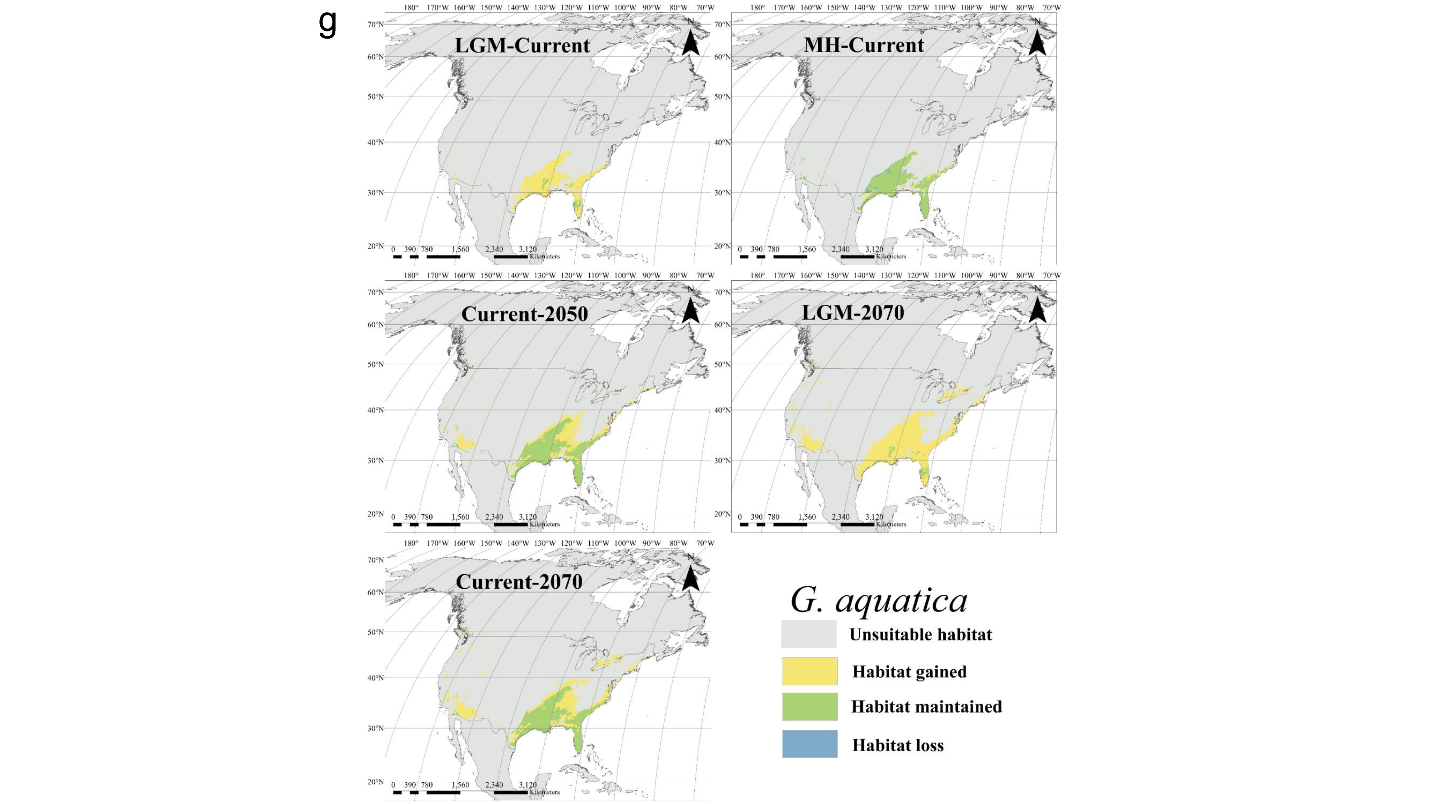


**Figure S7:** Area changes in the distribution area of *Gleditsia* at different time intervals. All species names are abbreviations for the genus *Gleditsia* (e.g., *G. aqustica* refers to *Gleditsia aqustica*). LGM and MH stand for the Last Glacial Maximum and the Mid-Holocene, respectively.


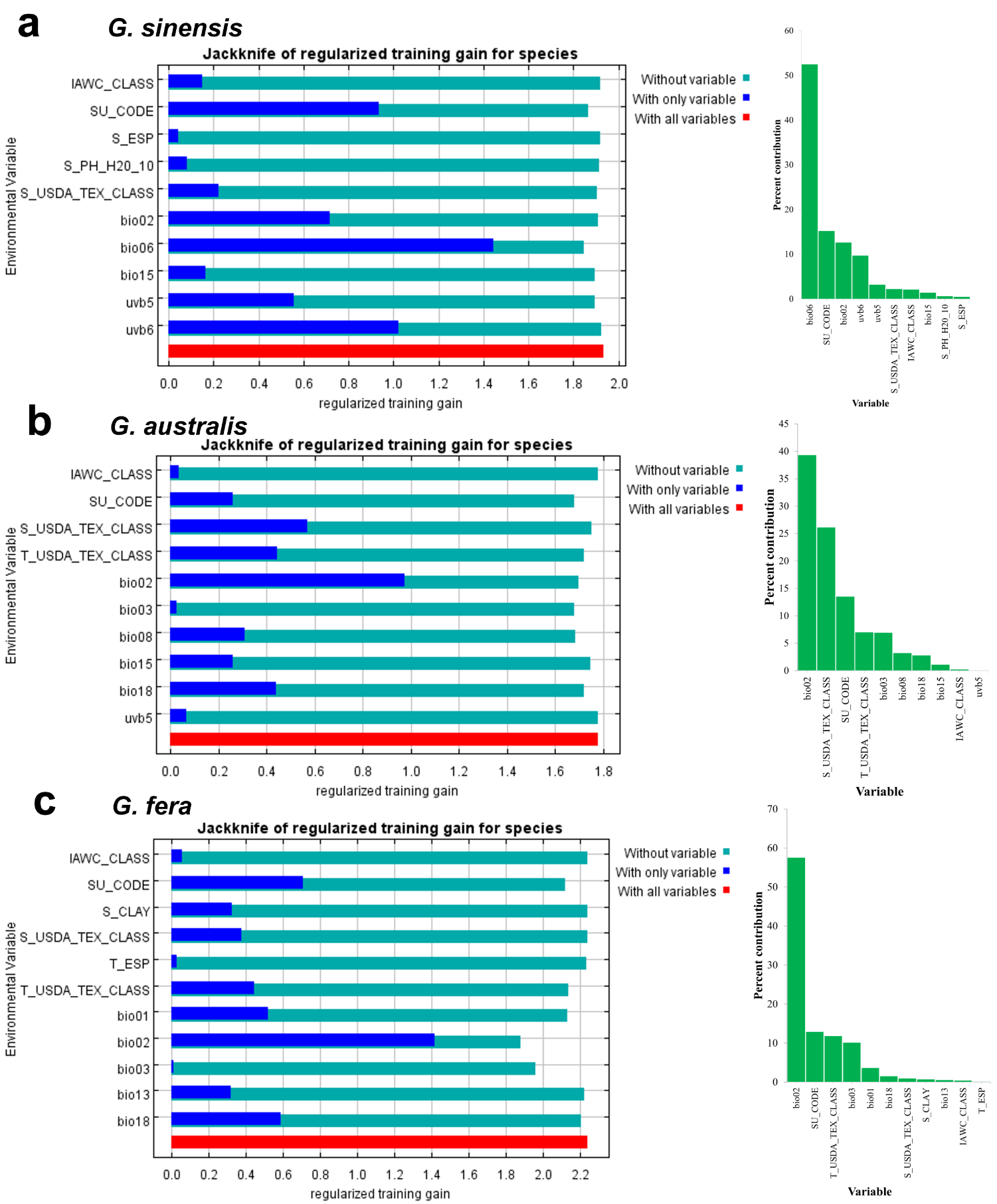


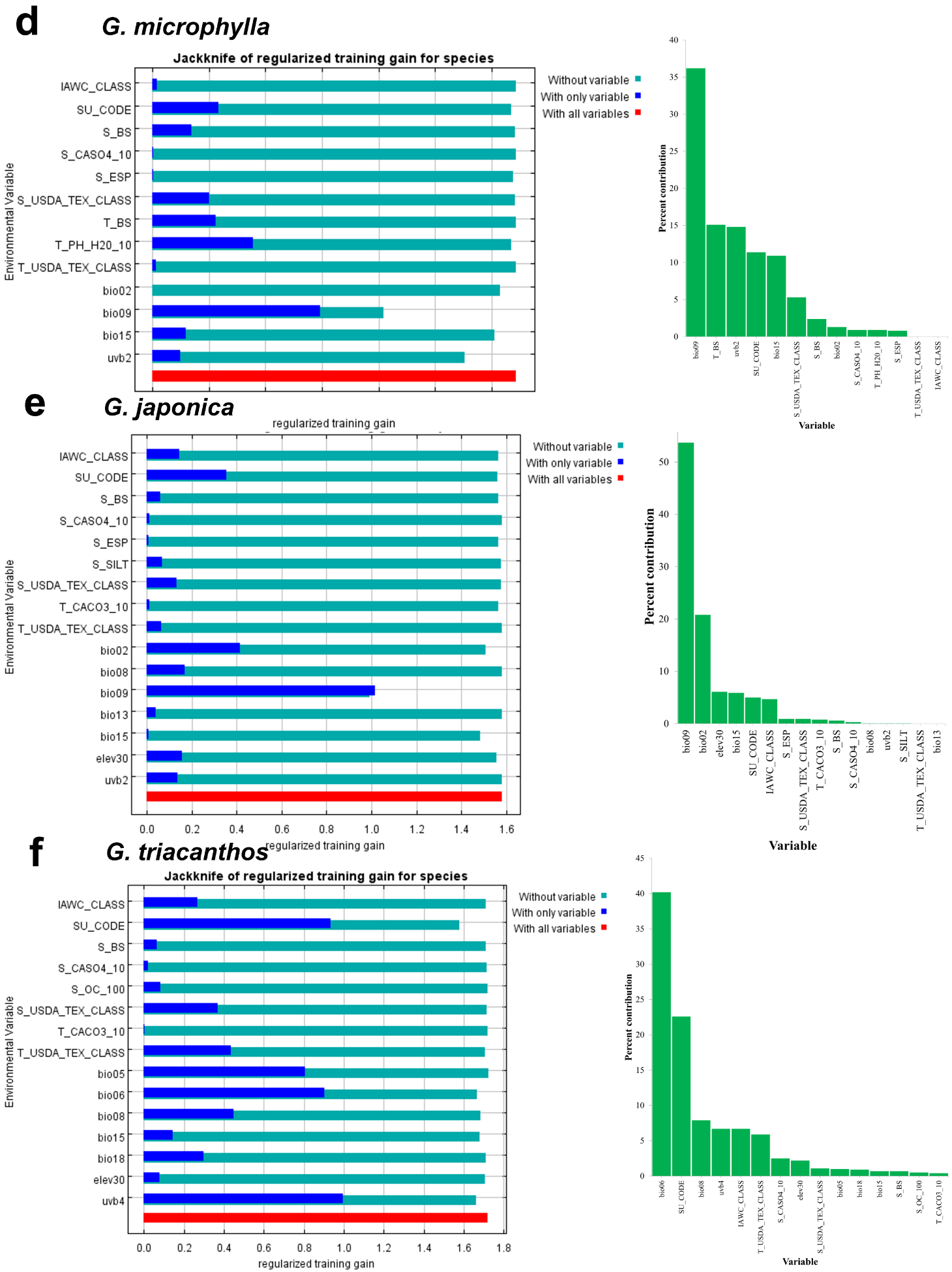


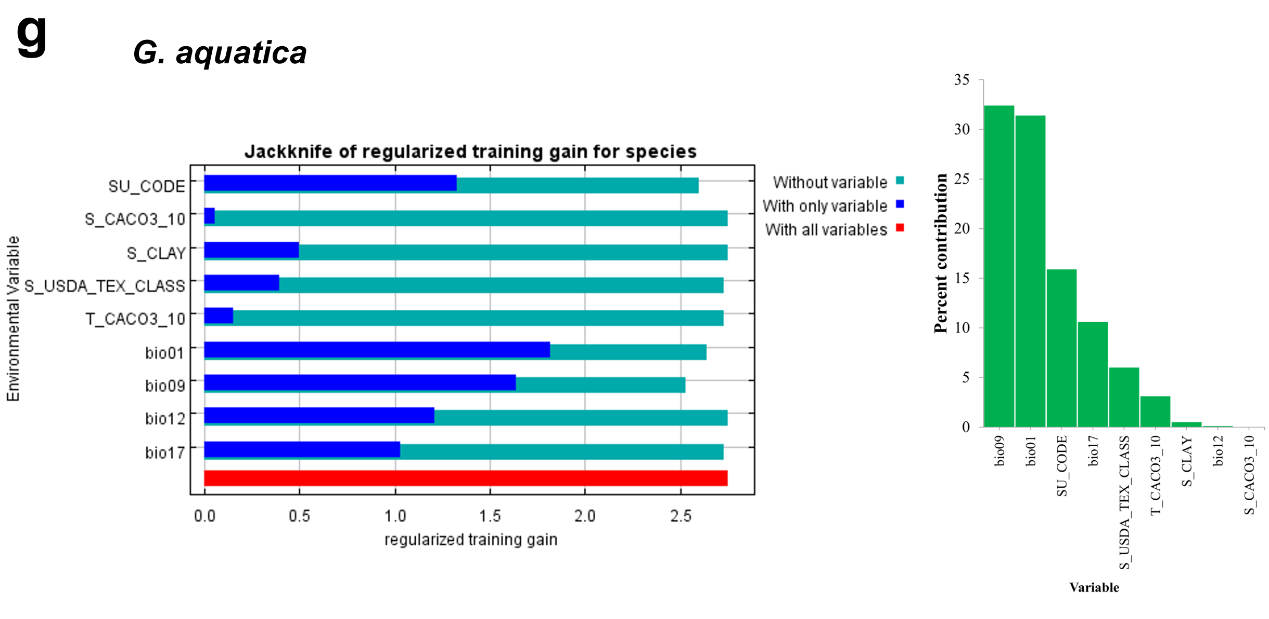


**Figure S8:** Jackknife test results and the percentage contribution for *Gleditsia.* All species names are abbreviations for the genus *Gleditsia* (e.g., *G. aqustica* refers to *Gleditsia aqustica*).


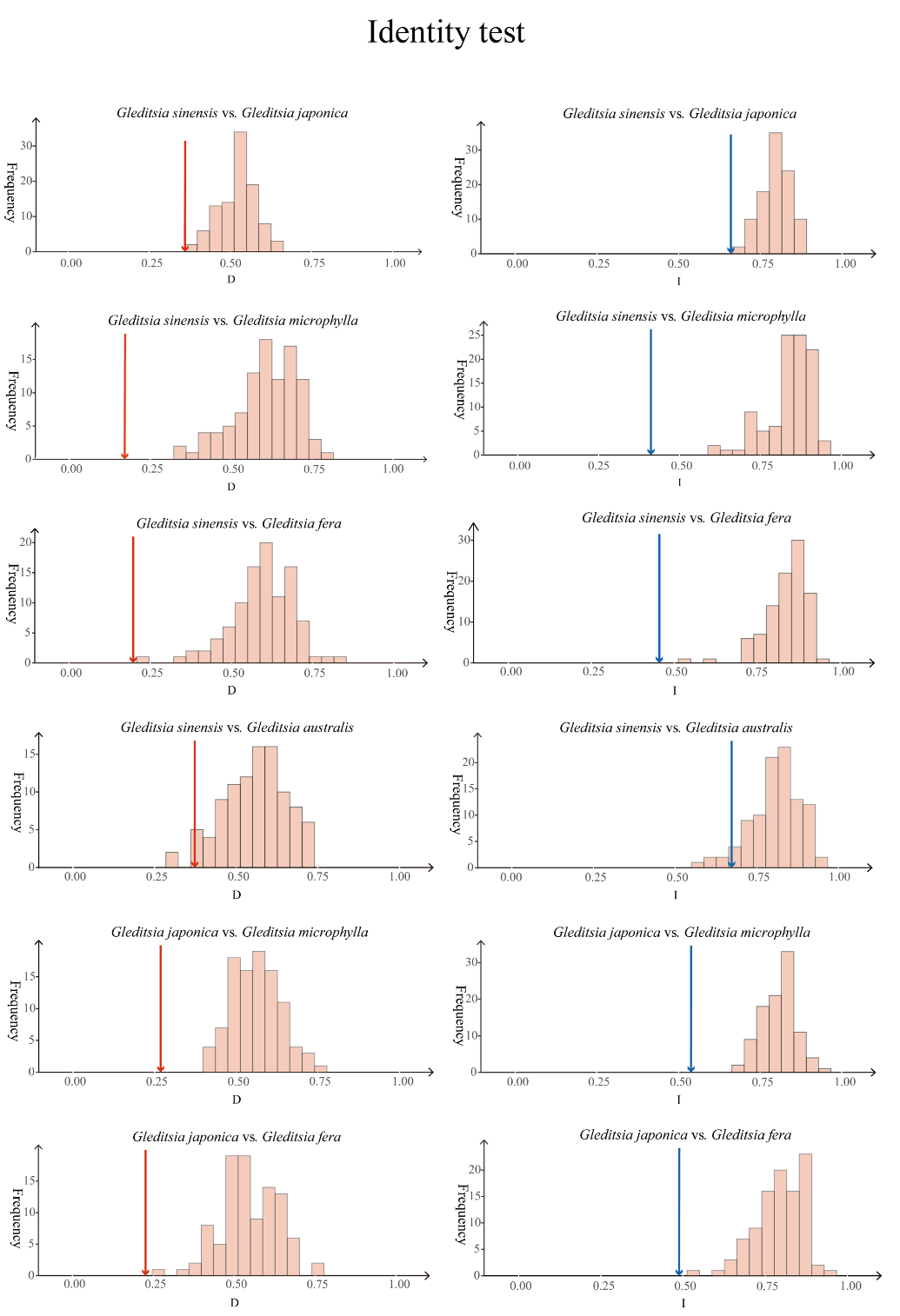


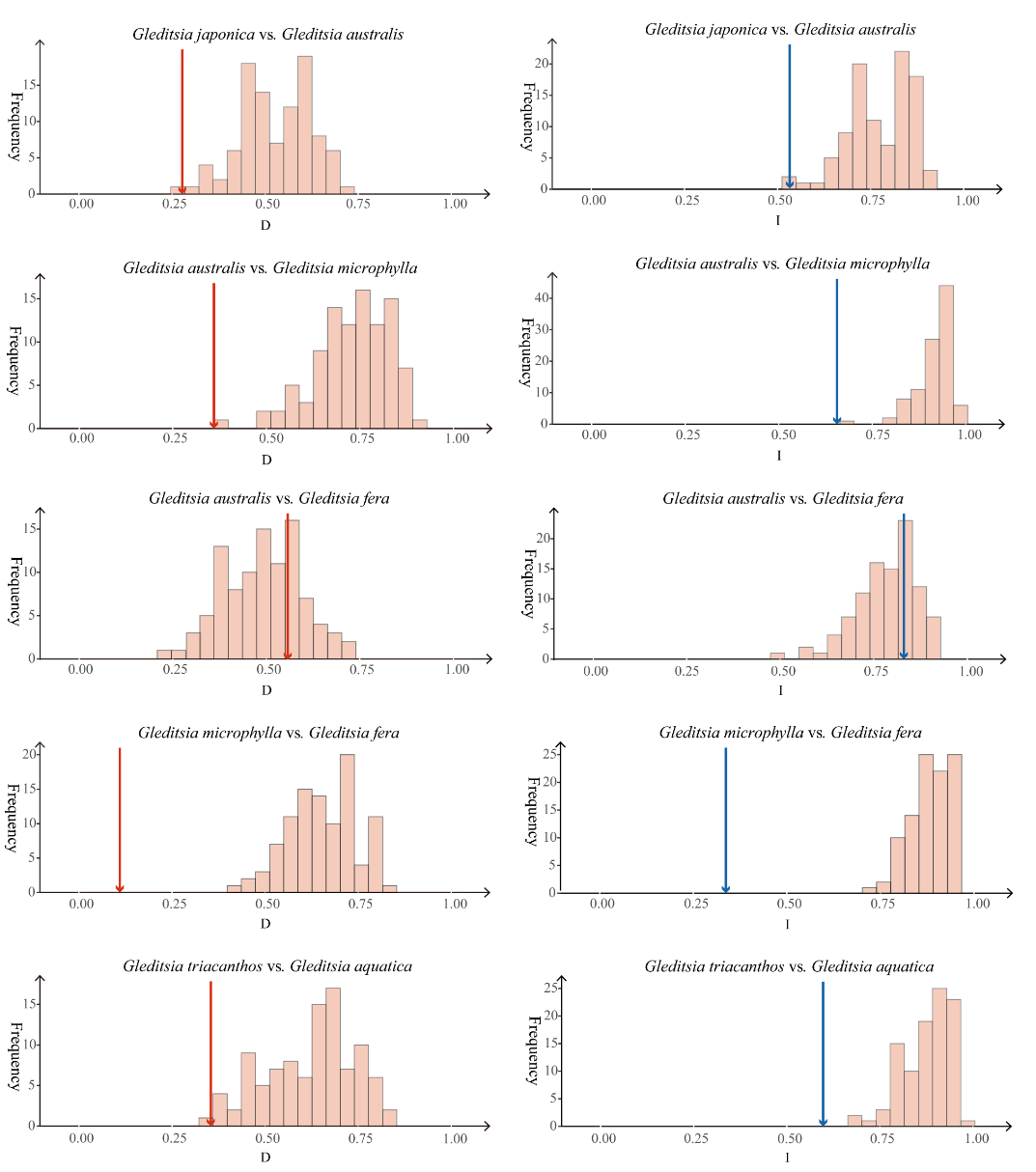


**Figure S9:** Sampling for identity testing. The environmental niches of paired species were compared. Arrows represent similarity scores of ENMs constructed for actual occurrences of two species (red for D, blue for I). The bar graph represents repeated randomization of the group identity (such as species or pedigree) for each data point to construct a null distribution. Both are generated from 1000 randomizations.


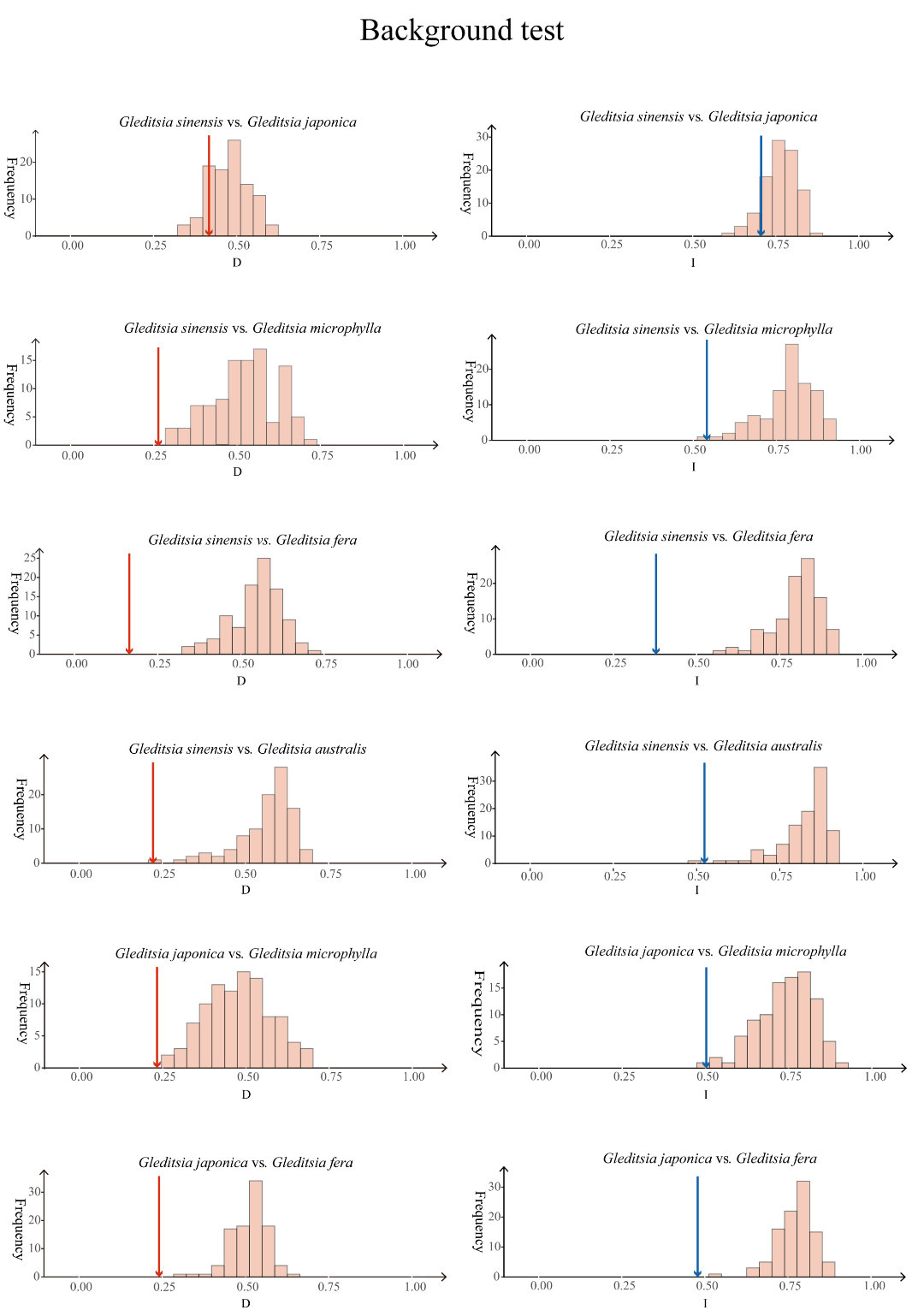


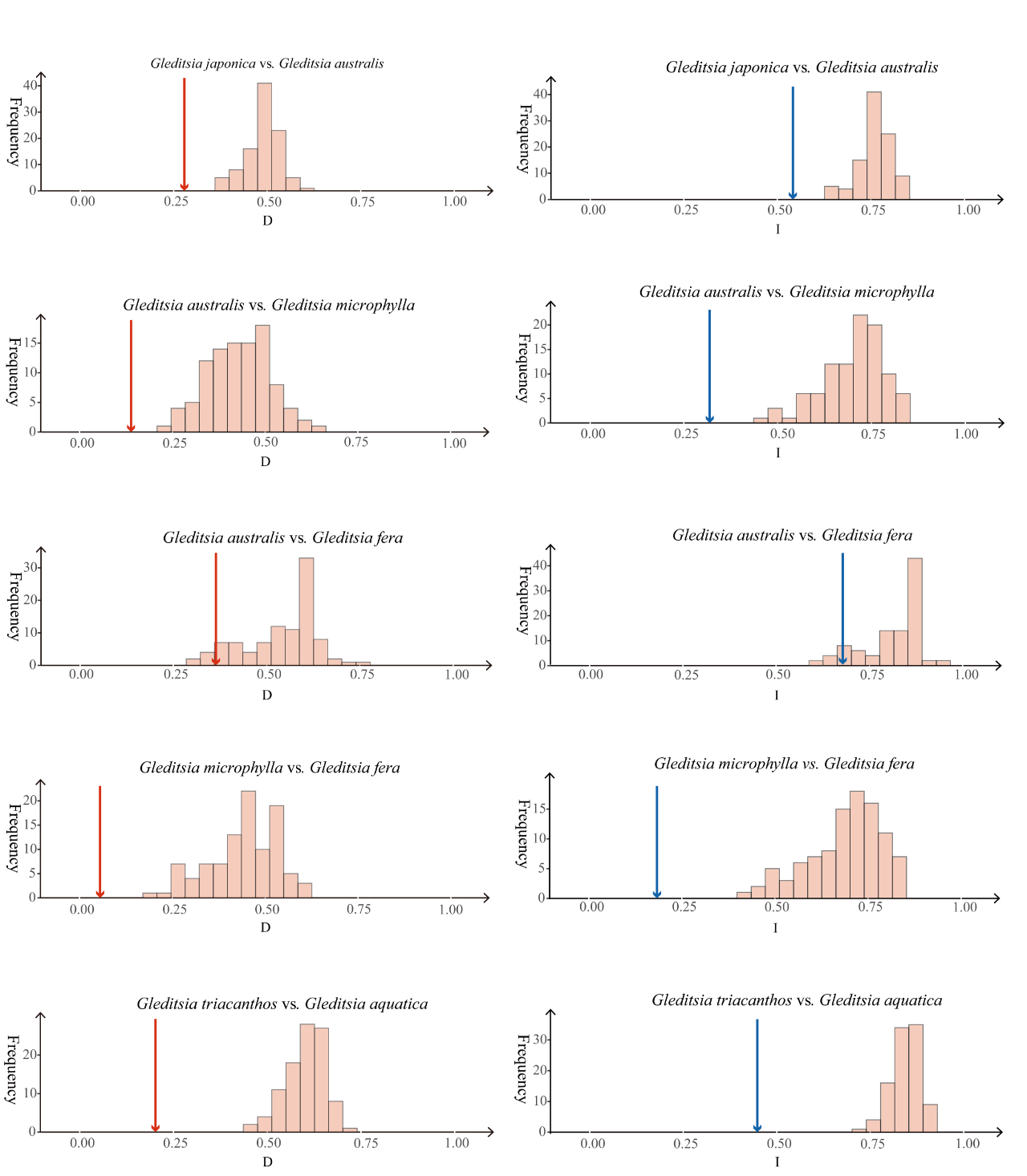


**Figure S10:** Sampling for background test. Background test sampling. Arrows represent similarity scores for ENMs constructed from known occurrences of two species (red for D, blue for I), and bars represent similarity scores for ENMs constructed using points randomly drawn from an area defined as the "environmental background" of one of the species. Randomly draw 1000 times.


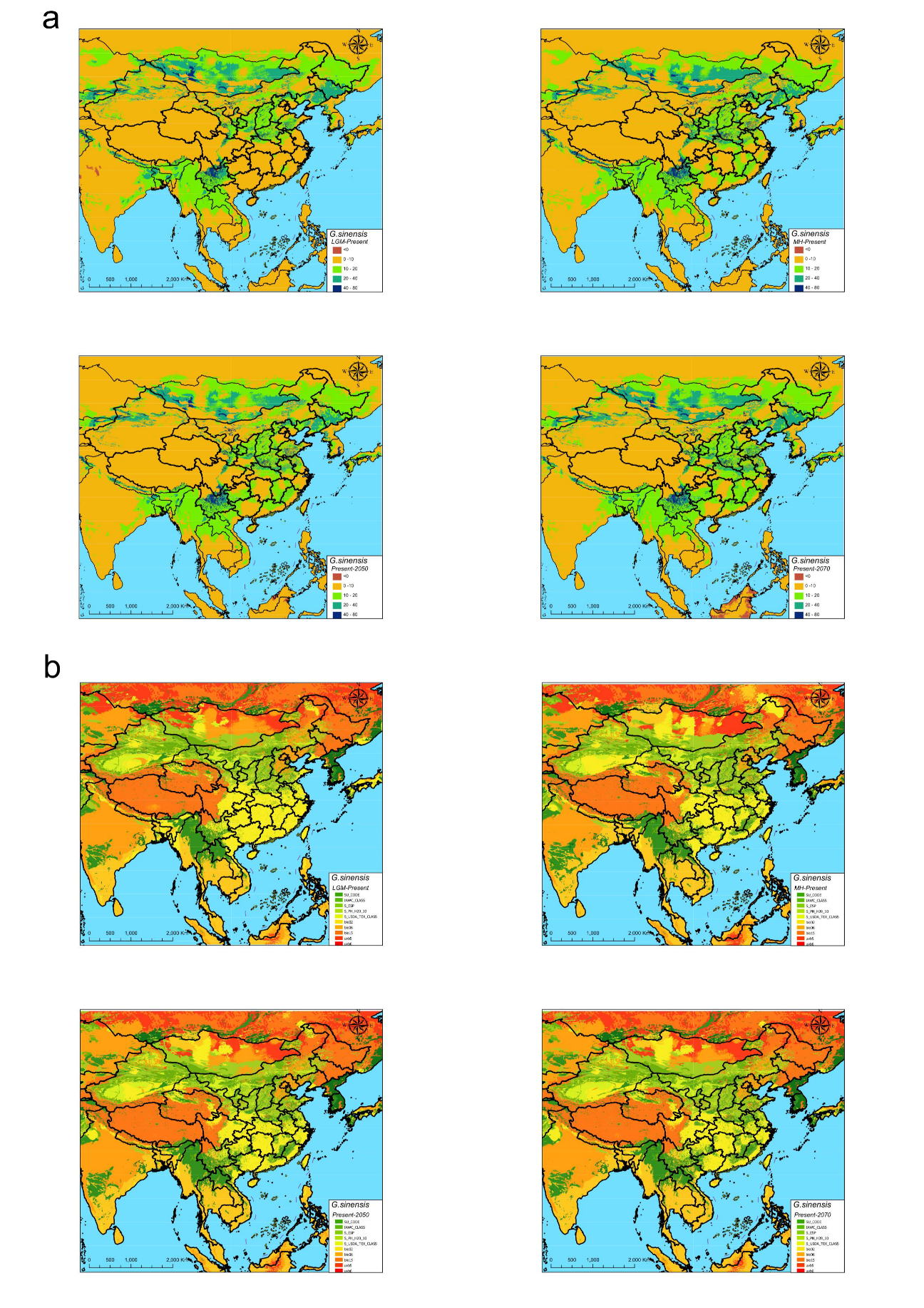


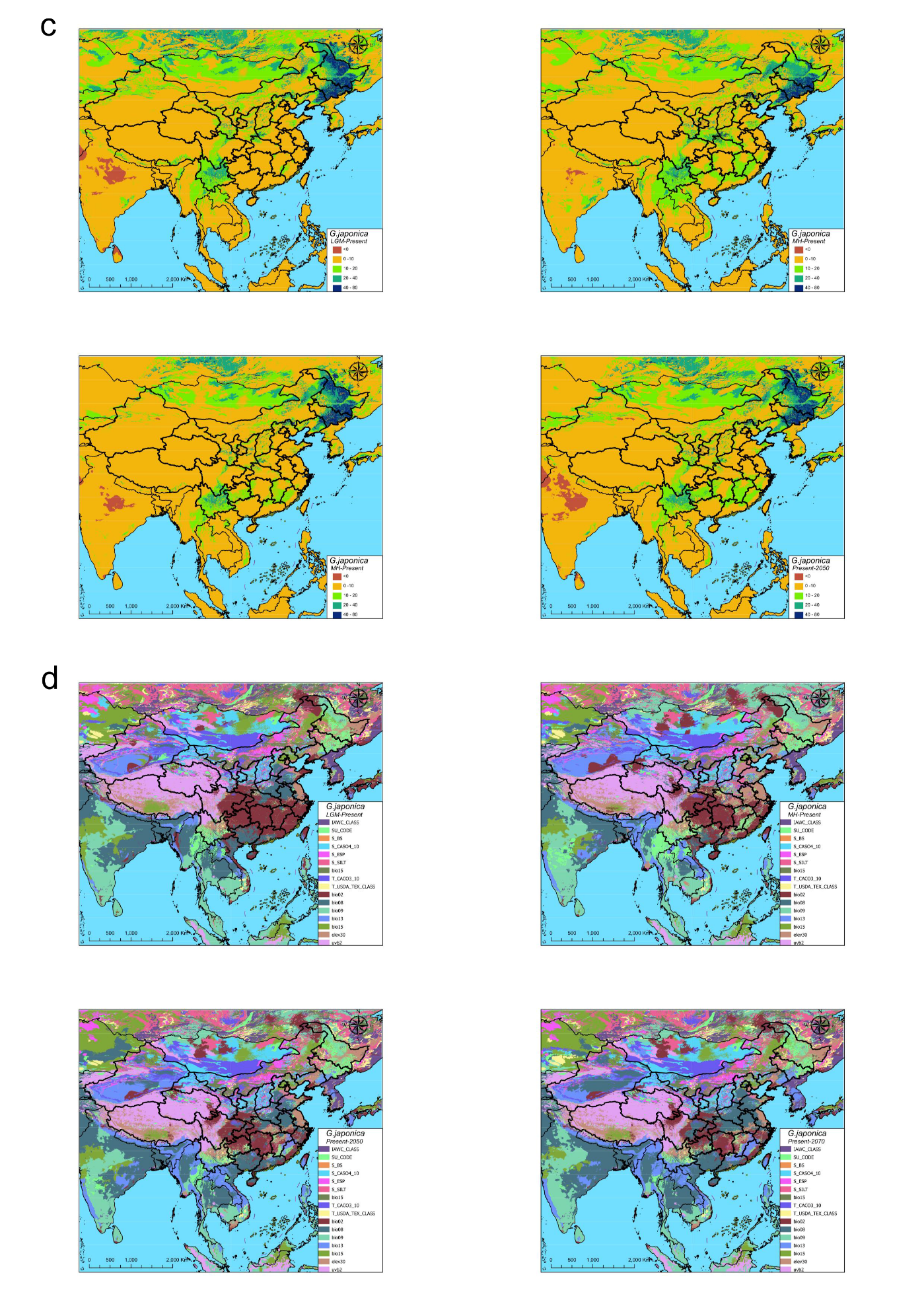


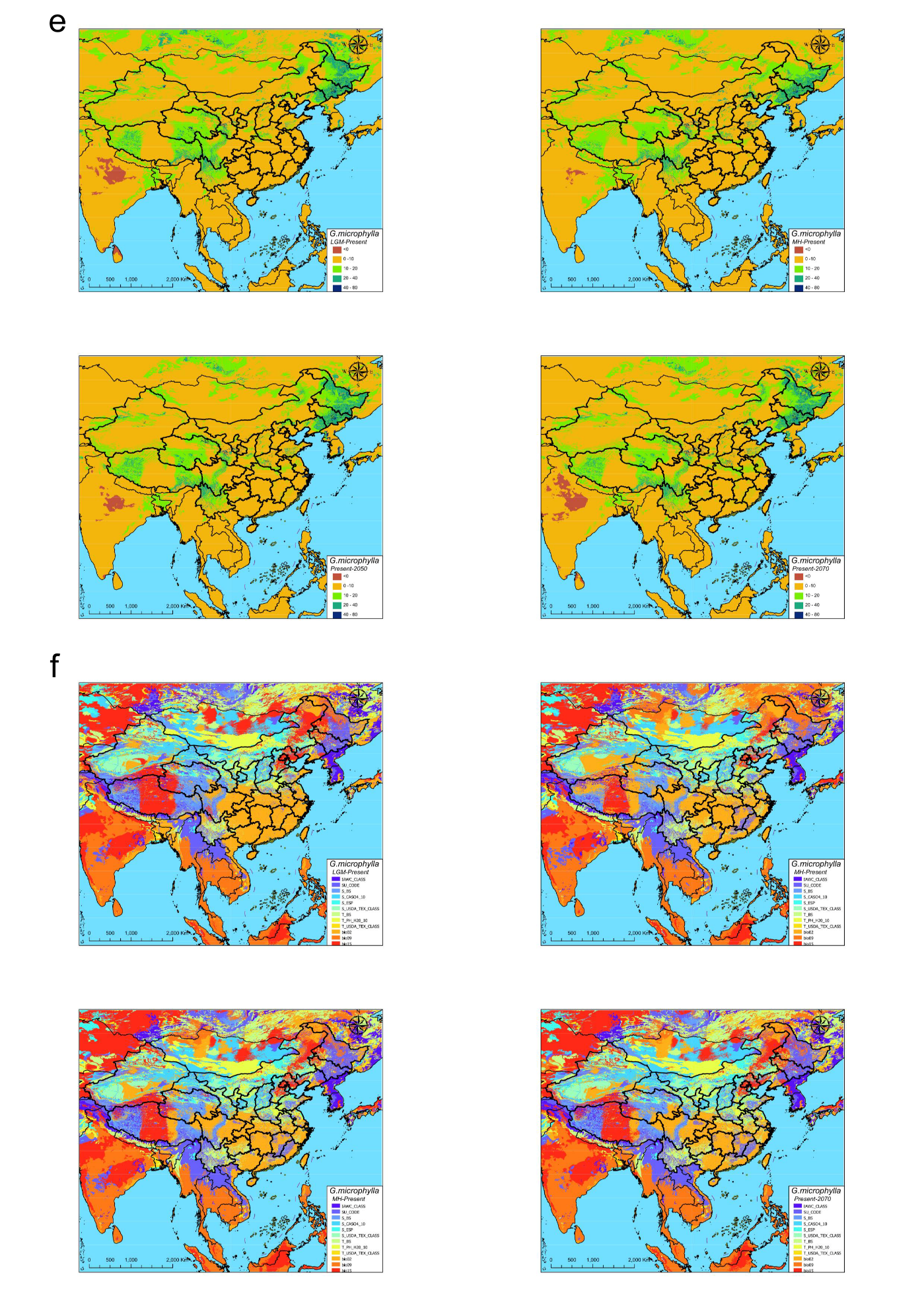


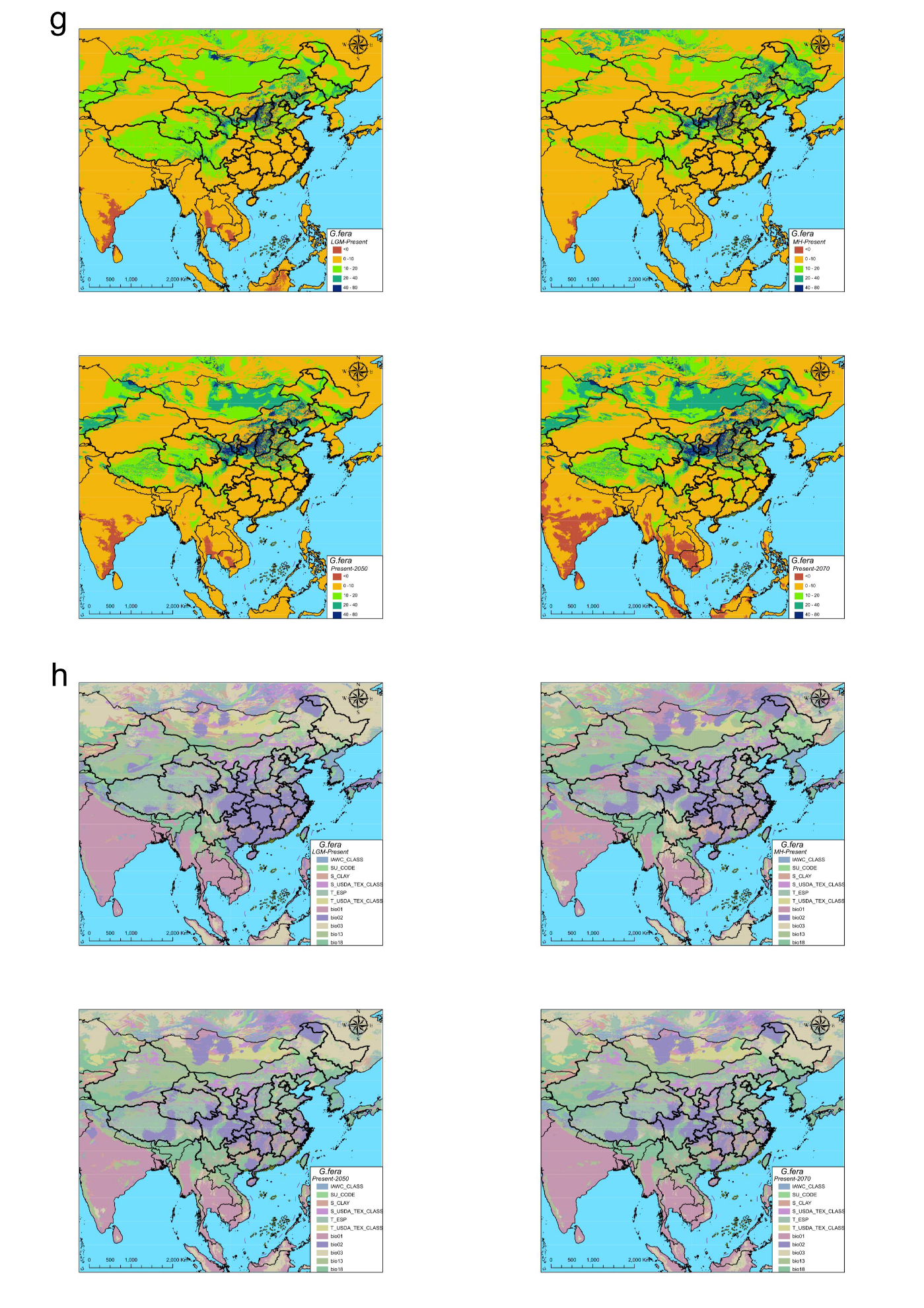


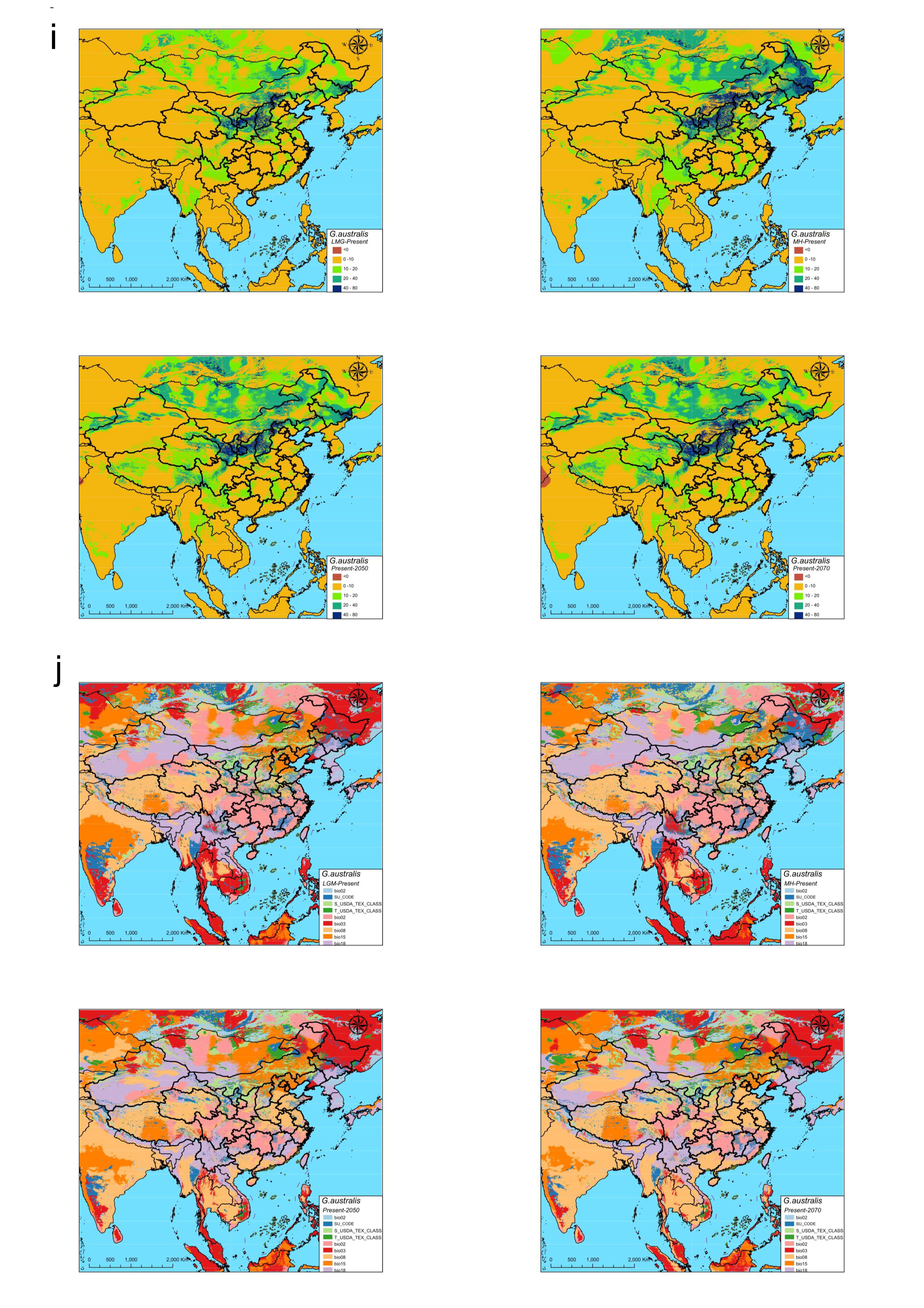

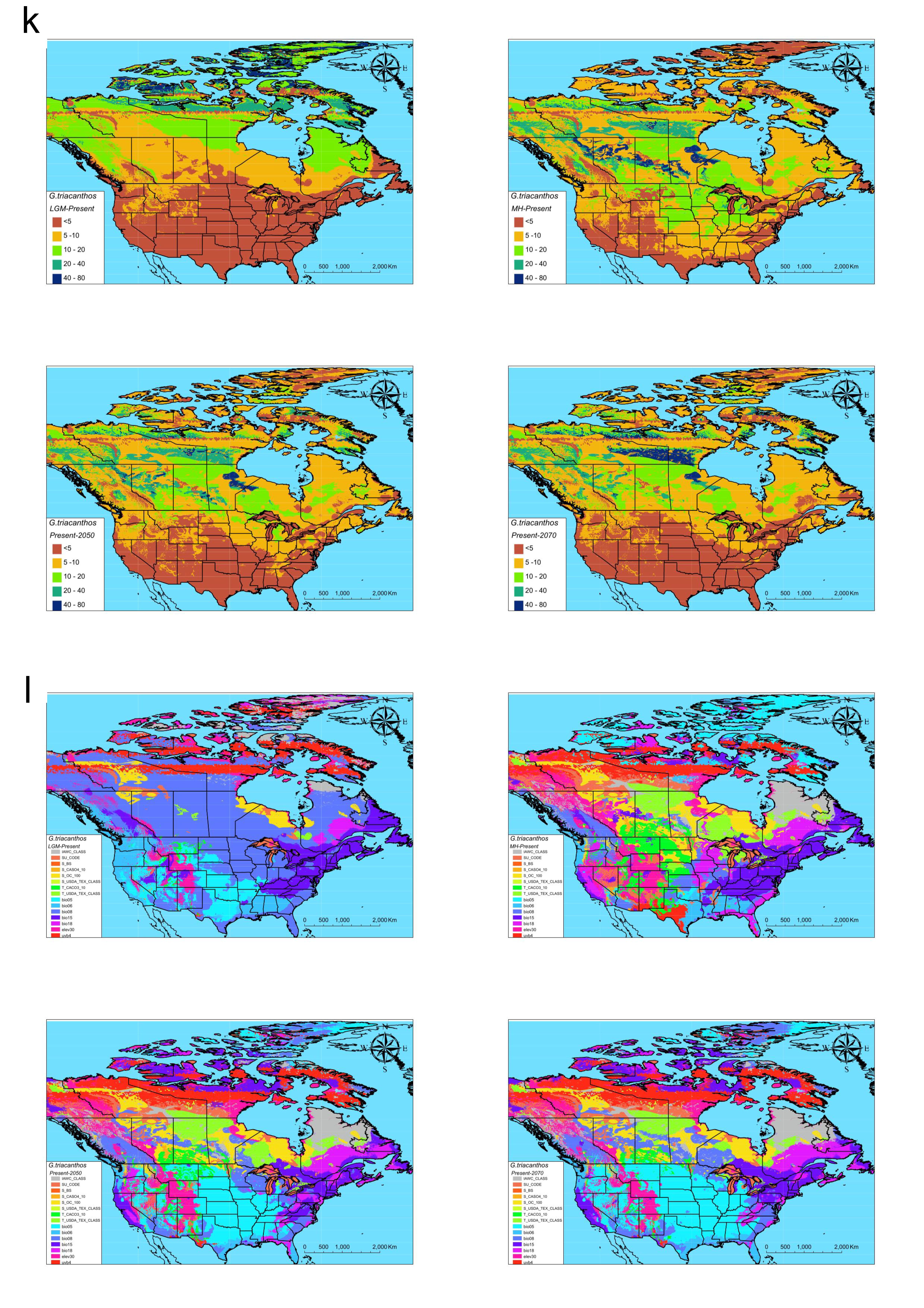

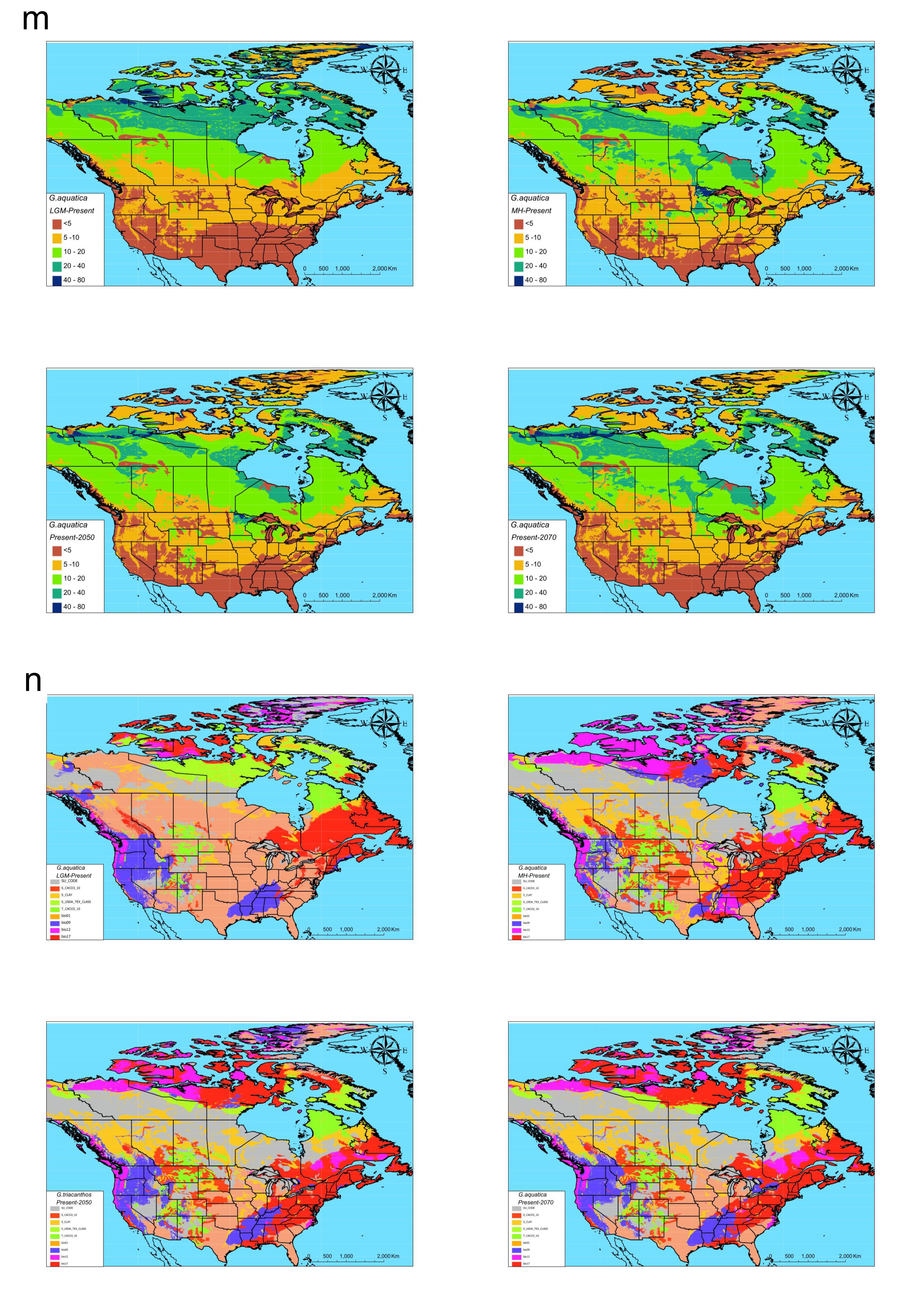


**Figure S11:** Multivariate environmental similarity surface (a, c, e, g, i, k, m) and least similar variable analysis (b, d, f, h, j, n). All species names are abbreviations for the genus *Gleditsia* (e.g., *G. aqustica* refers to *Gleditsia aqustica*). LGM and MH stand for the Last Glacial Maximum and the Mid-Holocene, respectively.
